# Morpho-biochemical characterization of a RIL population for seed parameters and identification of candidate genes regulating seed size trait in lentil (*Lens culinaris* Medik.)

**DOI:** 10.3389/fpls.2023.1091432

**Published:** 2023-02-15

**Authors:** Haragopal Dutta, Shivaprasad K. M., Muraleedhar S. Aski, Gyan P. Mishra, Subodh Kumar Sinha, Dunna Vijay, Manjunath Prasad C. T., Shouvik Das, Prashant Anupama-Mohan Pawar, Dwijesh C. Mishra, Amit Kumar Singh, Atul Kumar, Kuldeep Tripathi, Ranjeet Ranjan Kumar, Sanjeev Gupta, Shiv Kumar, Harsh Kumar Dikshit

**Affiliations:** ^1^ Division of Genetics, Indian Agricultural Research Institute, New Delhi, India; ^2^ Indian Council of Agricultural Research (ICAR)-National Institute for Plant Biotechnology, New Delhi, India; ^3^ Division of Seed Science and Technology, Indian Agricultural Research Institute, New Delhi, India; ^4^ Laboratory of Plant Cell Wall Biology, Regional Centre for Biotechnology, Faridabad, India; ^5^ Agricultural Bioinformatics, Indian Agricultural Statistics Research Institute, New Delhi, India; ^6^ Division of Genomic Resources, National Bureau of Plant Genetic Resources, New Delhi, India; ^7^ Germplasm Evaluation Division, National Bureau of Plant Genetic Resources, New Delhi, India; ^8^ Division of Biochemistry, Indian Agricultural Research Institute, New Delhi, India; ^9^ Krishi Bhawan, Indian Council of Agricultural Research, New Delhi, India; ^10^ South Asia and China Program, International Center for Agricultural Research in the Dry Areas, National Agriculture Science Complex (NASC) Complex, New Delhi, India

**Keywords:** BSA, cell membrane, cellulose, lignin, masur, videometer, xylose

## Abstract

The seed size and shape in lentil (*Lens culinaris* Medik.) are important quality traits as these influences the milled grain yield, cooking time, and market class of the grains. Linkage analysis was done for seed size in a RIL (F_5:6_) population derived by crossing L830 (20.9 g/1000 seeds) with L4602 (42.13 g/1000 seeds) which consisted of 188 lines (15.0 to 40.5 g/1000 seeds). Parental polymorphism survey using 394 SSRs identified 31 polymorphic primers, which were used for the bulked segregant analysis (BSA). Marker PBALC449 differentiated the parents and small seed size bulk only, whereas large seeded bulk or the individual plants constituting the large-seeded bulk could not be differentiated. Single plant analysis identified only six recombinant and 13 heterozygotes, of 93 small-seeded RILs (<24.0 g/1000 seed). This clearly showed that the small seed size trait is very strongly regulated by the locus near PBLAC449; whereas, large seed size trait seems governed by more than one locus. The PCR amplified products from the PBLAC449 marker (149bp from L4602 and 131bp from L830) were cloned, sequenced and BLAST searched using the lentil reference genome and was found amplified from chromosome 03. Afterward, the nearby region on chromosome 3 was searched, and a few candidate genes like ubiquitin carboxyl-terminal hydrolase, E3 ubiquitin ligase, TIFY-like protein, and hexosyltransferase having a role in seed size determination were identified. Validation study in another RIL mapping population which is differing for seed size, showed a number of SNPs and InDels among these genes when studied using whole genome resequencing (WGRS) approach. Biochemical parameters like cellulose, lignin, and xylose content showed no significant differences between parents and the extreme RILs, at maturity. Various seed morphological traits like area, length, width, compactness, volume, perimeter, etc., when measured using VideometerLab 4.0 showed significant differences for the parents and RILs. The results have ultimately helped in better understanding the region regulating the seed size trait in genomically less explored crops like lentils.

## Introduction

Lentil (*Lens culinaris* ssp. *culinaris* Medik.) is a diploid (2n=14), self-pollinated, cool season legume crop having a genome size of nearly 4.2 Gb (Arumuganathan and Earle, 1991; Dikshit et al., 2022a; Dikshit et al., 2022b). This is not only rich in proteins but also in micronutrients (Fe and Zn) and β-carotene (Mishra et al., 2020; Priti et al., 2021; Priti et al., 2022). Lentil is being grown throughout the world in temperate to sub-tropical regions including regions of the Middle East, north-eastern Africa, Southern Europe, South and North America, Australia, and the Indian sub-continent (Mishra et al., 2022a). Globally, Canada is the largest producer and exporter of lentils. Lentil is an important crop for India having acreage of 1.35 m ha and production of 1.18 m tons. The world production of lentils is 6.54 m tons from an area of nearly 5.01 m ha. Lentil productivity in India (871.5 kg/ha) is well below world productivity (1304.9 kg/ha) (FAOSTAT, 2020).

Seed quality of lentil is an important objective for both industry and the consumer. Among various parameters, seed size is the key parameter defining the overall lentil quality (Singh et al., 2022). During domestication of lentils, several traits like pod dehiscence, dormancy, and seed size got modified which ultimately allowed easy collection of seeds by the farmers for next year sowing (Sonnante et al., 2009). Most of the domestication traits like pod dehiscence, dormancy, and growth habit are single gene governed traits while seed size is a quantitative trait. Depending upon the seed size lentil is classified into microsperma type (2 to 6 mm diameter, red and yellow cotyledons, and pigmented flowers) and macrosperma type (6 to 9 mm diameter, yellow cotyledon, and non-pigmented flowers) (Barulina, 1930; Sandhu and Singh, 2007). Generally, microsperma types are more common in southeast Asia, while macrosperma types in western Asia and Europe (Barulina, 1930). Previous genetic studies revealed large variations for seed weight and seed diameter in lentils (Tullu et al., 2001; Dutta et al., 2022; Tripathi et al., 2022).

Seed size and shape are known to influence both cooking time and dehulling efficiency and are considered important market-associated trait (Erskine et al., 1991; Wang, 2008). A strong positive correlation (r=0.96) was recorded between seed size and cooking time (Hamdi et al., 1991). Ford et al. (2007) noted reduced damage during handling in the rounder seed-shaped lentil cultivars over thin, sharp-edged types. Thus, the development of genotypes with improved seed parameters including seed weight is an important breeding objective of lentil breeders across the globe (Mishra et al., 2022b). Generally, seed parameters are measured using crude phenotypic evaluation methods like measurement of 100-grain weight or seed diameter measurement using Vernier caliper or graded sieve (Hossain et al., 2010; Xu et al., 2011). In lentils, seed diameter was also measured using computer-assisted 2-dimensional imaging (Shahin and Symons, 2001) and seed plumpness was determined using 3-dimensional imaging using a camera (Shahin et al., 2006). However, these are laborious methods, especially when a large number of genotypes are involved in screening. Recently, Dutta et al. (2022) used a very easy method involving VideometerLab 4.0 instrument for the measurement of various seed parameters in lentils.

Linked molecular markers with the trait of interest will help in efficient breeding for that trait (Mishra et al., 2003). Seed weight is known to be governed by several genes and thus identification of linked markers with the seed weight QTLs will help in the better selection for this trait. This will also help in speeding up of new variety development having desired seed parameters (Tripathi et al., 2022). Also, for the implementation of molecular breeding approach for seed size trait, there is a need for the development of an experimental population involving contrasting parents, so that the linkage can be established between marker and the trait. Evaluation of a RIL population with SSRs markers using BSA approach can help in the identification of linked markers with the seed size trait in lentil (Mishra et al., 2001; Mishra et al., 2003). This study hypothesizes that the genomic region controlling the seed size can be identified using molecular markers in a mapping population differing for the seed size trait. Against this backdrop, the objective of this study was to perform the morpho-biochemical characterization of a RIL population for seed parameters and identification of candidate genes regulating seed size trait in lentil.

## Materials and methods

### Plant materials

**Figure 1:**
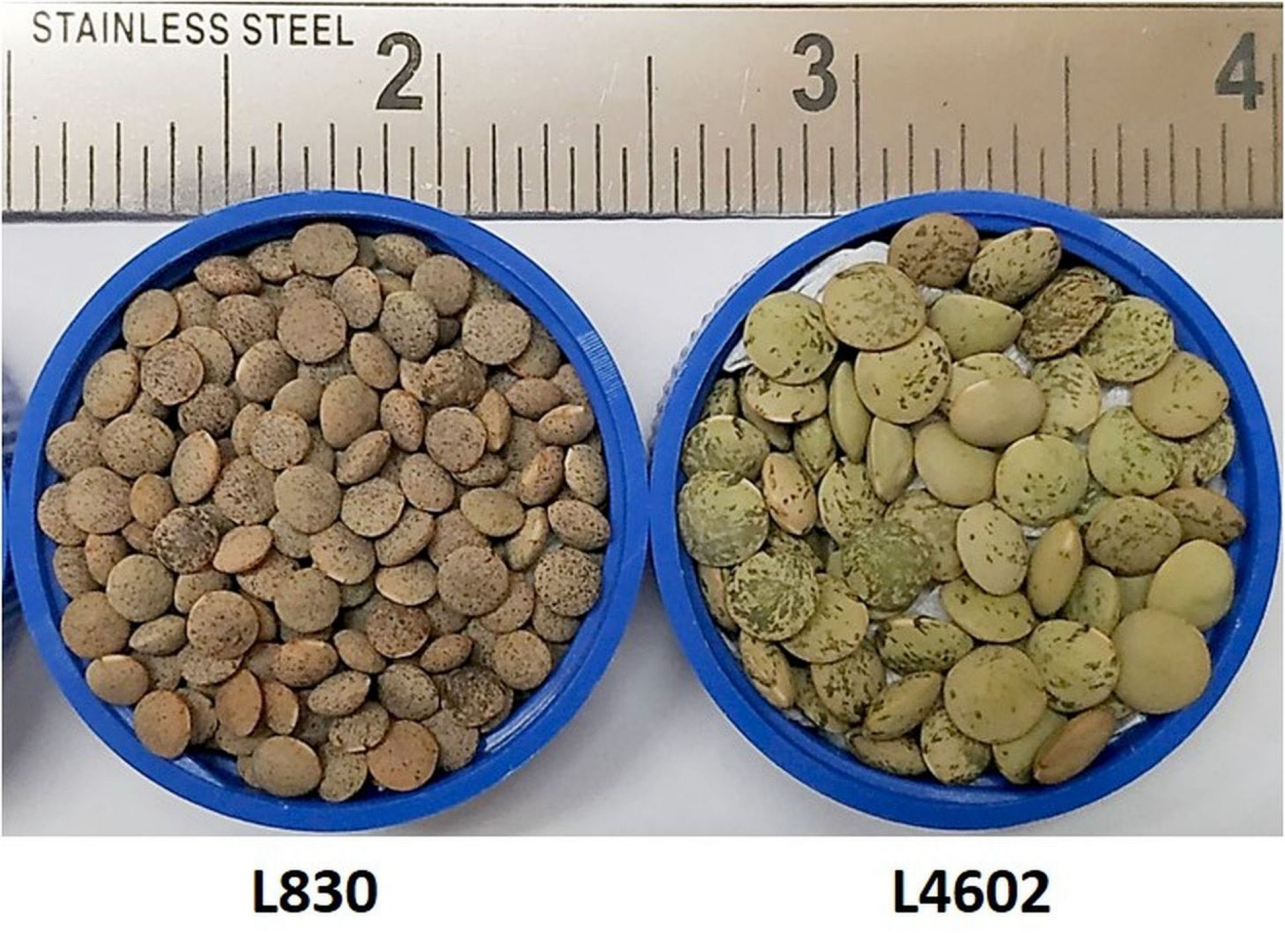
Seeds of the small-seeded (L830, mean 1000 seed weight=20.9g) and large-seeded (L4602, mean 1000 seed weight=42.13g) parents were used for the seed size analysis.

**Figure 2:**
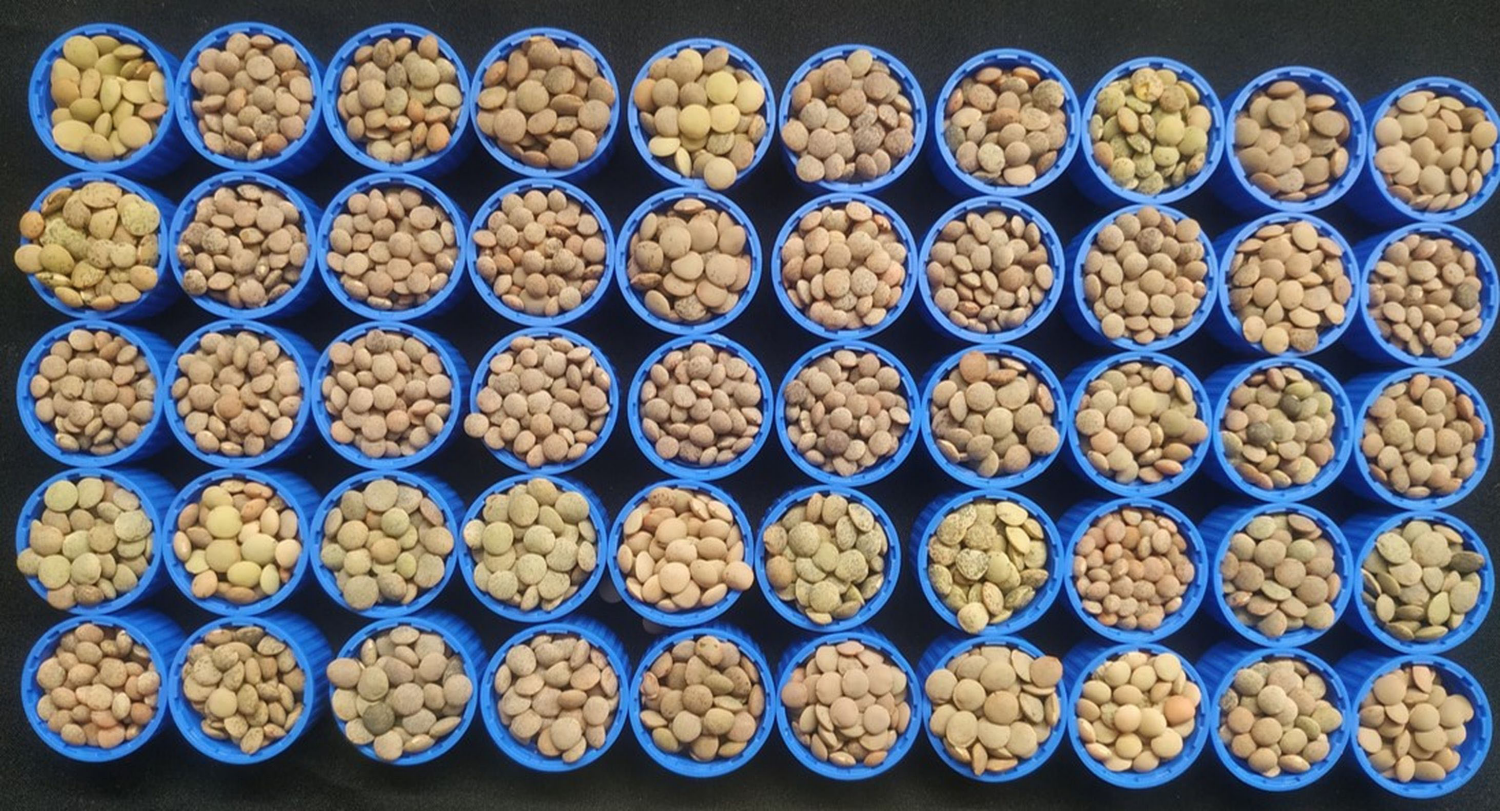
A representative figure showing variations for the seed parameters in a set of 50 RILs (F_5:6_), derived from the cross L4602×L830 (mean 1000 seed weight range=15.0 to 40.5g).

Two lentil genotypes differing significantly in seed size, L830 (small-seeded; mean 1000 seed weight = 20.9 g) and L4602 (large-seeded; mean 1000 seed weight = 42.13g) were used as the parent for the development of a RIL population (**Figure 1**). Cross was attempted between the L4602 and L830 and the F_1_ was confirmed for its hybridity using polymorphic SSR markers. The parents and the RIL population (F_5:6_) having 188 lines were grown during rabi-2021 at the fields of Indian Agricultural Research Institute, New Delhi, India (Latitude: 28.6412°N, Longitude: 77.1627°E, and Altitude: 228.61 m AMSL) with the spacing of 30×5 cm (row to row × plant to plant) in a 5.0 m row length using standard cultivation practices. Each row was harvested at maturity and 1000 seed weight was measured for parents and the RILs (**Figures 2**, **S1**).

### DNA extraction and constitution of bulks for bulked segregant analysis

**Figure 3:**
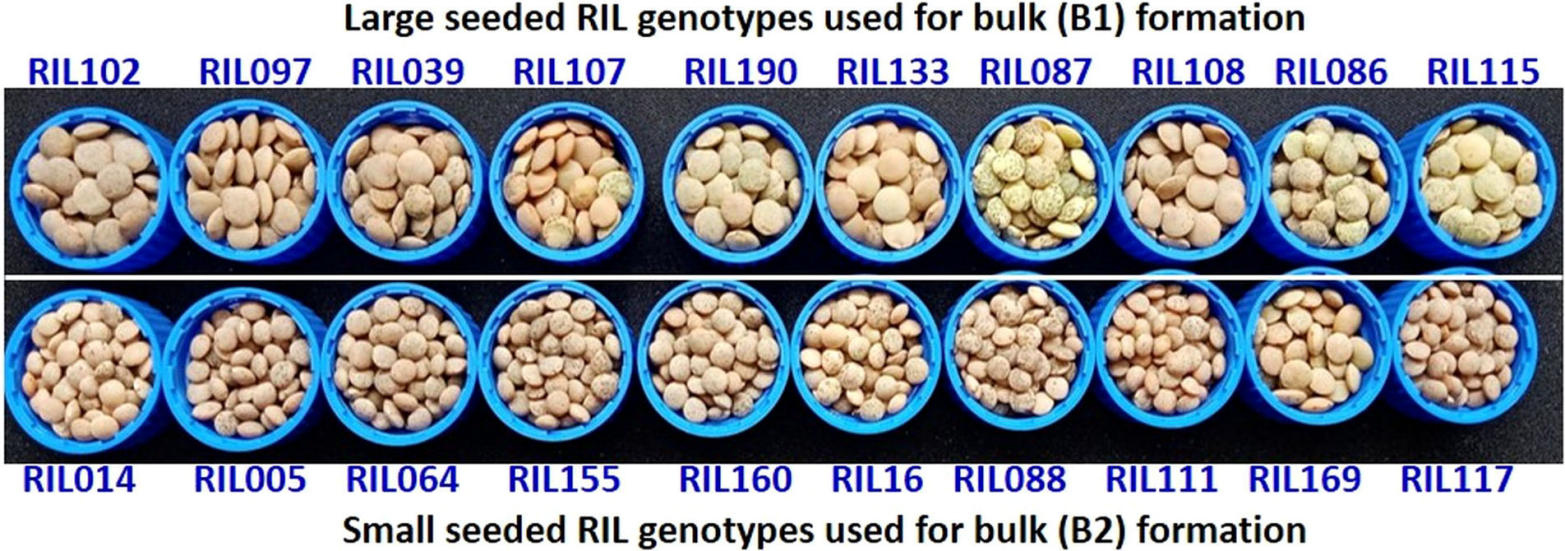
The 10 extreme RIL genotypes for seed size which was used for the formation of two extreme bulks for the BSA. Where, upper panel represents 10 RILs with maximum seed size (in descending order of their seed weight) and lower panel represents 10 RILs with minimum seed size (in ascending order of their seed weight).

Nearly 15-20 seeds each from 188 RILs along with the parents (L830 and L4602) were kept on the germination paper and was wrapped in a butter paper. This was then kept in a germination chamber for 8-10 days at 20-25°C. The tender seedlings were used for DNA isolation using CTAB method (Murray and Thompson, 1980) and quality was checked on 0.8% Agarose gel, while quantity was measured using Nanodrop (García-Alegría et al., 2020). A total of 10 extreme genotypes each from small-seeded RILs (line No. 05, 14, 16, 64, 88, 111, 117, 155, 160, 169) and large-seeded RILs (line No. 03, 86, 87, 97, 102, 107, 108, 115, 133, 190) were used for the BSA (**Figure 3**). An equal quantity of DNA (20 ng/µL) was taken from each of the 10 extreme RILs and mixed to constitute the two contrasting bulks (B1 and B2). A total of 394 SSRs were used for the parental polymorphism survey (**Table S1**) and polymorphic primers were used for BSA (Michelmore et al., 1991) and band were separated on 3.0% Metaphor agarose gel and scored. The SSRs differentiating the bulks and the parents were used for the individual RIL analysis. The RILs were arranged in the increasing order of their seed size, PCR was performed and amplification was visualized on the gel using gel documentation system.

### Cloning and sequencing of a PCR amplified product

The DNA fragment amplified by an SSR marker (PBALC449) in L4602 and L830 was used for the cloning and sequencing. The amplified bands containing the DNA were first precisely excised from the gel with a clean, sharp scalpel and then DNA was extracted using QIAquick Gel Extraction Kit (QIAGEN, Valencia, USA) by following the manufacturer’s instructions (Sambrook et al., 1989). The amplified product was ligated with pJET1.2/blunt vector using CloneJET PCR Cloning Kit (Thermo Fisher Scientific™) as per the mentioned protocol (https://www.thermofisher.com/document-connect/document-connect.html). Then the recombinant vector was transformed into *E. coli* DH5α strain competent cells for cloning using the standard protocol. Afterward, plasmid was isolated using FavorPrep Plasmid Extraction Mini Kit as per the manufacturer’s instructions and extracted plasmid DNA was stored at -20°C for further analysis. The cloning was confirmed by restriction digestion using *Bgl* II. The positive clones were sequenced using Sanger sequencing platform using universal primer. The raw sequence data was processed by trimming the vector sequence and aligned to the reference genome (CDC Redberry Genome Assembly v2.0; Ramsay et al., 2021) using NCBI BLAST browser.

### Biochemical analysis of lentil genotypes and the extreme RILs differing for seed size

Various cell wall related biochemical analyses were performed on the 10 extreme RILs each for seed size (large and small seeded RILs), and the parents.

### Preparation of alcohol insoluble residue sample

Briefly, 600 mg of lentil seeds were crushed, flash frozen (in liquid N_2_), and ground in Qiagen TissueLyser II (at 30 Hz for 2-3 min) to a fine powder. Then 100 mg powder was taken for incubation (at 70°C for 30 min) in 5.0 mL ethanol (80%) having 4.0 mm HEPES buffer (pH 7.5). This was then cooled on ice and centrifuged (1000 rpm for 15.0 min), supernatant was discarded, residue was washed (5.0 mL 70% ethanol) and then suspended in chloroform: methanol (1:1) solution (5.0 mL) for 3.0 min at room temperature and centrifuged (14000 rpm for 15.0 min). The residue was again washed with acetone (5.0 mL), pellet was dried in a desiccator and used as an AIR sample for further analysis (Pawar et al., 2017).

### Estimation of cellulose by Updegraff method

To the AIR sample (2.0 mg), Updegraff reagent (acetic acid: nitric acid: water = 8:1:2 v/v) was added and incubated (at 100°C for 30 min). The mixture was then centrifuged (10,000 rpm; 15 min), and pellet was washed four times with acetone and dried overnight. The dried residue was hydrolyzed in 72% H_2_SO_4_, glucose was analyzed by anthrone assay (Updegraff, 1969) and a standard curve was used to estimate the cellulose content.

### Estimation of xylose and O-acetyl content

AIR sample (2.0 g) was incubated for neutralization with HCl and NaOH for xylose and acetyl content estimation, respectively. The xylose and O-acetyl content were analyzed using Megazyme K-ACET and K-XYLOSE kits, respectively (Rastogi et al., 2022).

### Acetyl bromide soluble lignin content

The 25% acetyl bromide solution was diluted using acetic acid and incubated at 50°C for 2.0h. The solubilized powder was mixed with NaOH and hydroxylamine hydrochloride and then absorbance was recorded at 280 nm and lignin content was measured (Foster et al., 2010).

### Lignin and cellulose estimation through fourier transform-infrared spectroscopy

Lignin and cellulose contents were estimated using FTIR spectroscopy in the lentil seed powder (Pawar et al., 2017). A Tensor FTIR spectrometer (Bruker Optics) equipped with a single-reflectance horizontal ATR cell (ZnSe Optical Crystal, Bruker Optics) was used for the analysis. The spectrum range selected was from 600 cm^-1^ to 4000 cm^-1^ having a resolution of 4 cm^-1^. KBr powder was used for the preparation of standard and each sample was measured twice (by removing and adding different aliquots of powder for heterogeneity evaluation) and each spectrum was the average of 16 scans (Labbe et al., 2005; Canteri et al., 2019).

### Estimation of seed morphological parameters using VideometerLab 4.0 instrument

Detailed seed phenotyping was done for eight RILs (four large and small-seeded each) and the parents using VideometerLab 4.0 instrument (Videometer A/S, Denmark) which captured the images of 30 seeds placed in a customized 3D printed plate. Videometer acquires morphological and spectral information using 19 high power LED sources (375, 405, 435, 450, 470, 505, 525, 570, 590, 630, 645, 660, 700, 780, 850, 870, 890, 940, 970 nm). The data were quantified using custom-designed software (VideometerLab software ver. 2.13.83) as seed area, length, width, etc. (Shrestha et al., 2015).

### Statistical analysis

ANOVA was performed to determine the genotypic variance among parents and the 188 RIL genotypes (for various seed parameters like seed weight, area, length, width, width/length, compactness, width/area, volume, and perimeter) and also among the parents and the 10 extreme RIL genotypes (for biochemical parameters like lignin, cellulose, and xylose contents) using DSAASTAT ver.1.514 software. Afterward, multiple comparison test was performed using Tukey HSD method (p ≤ 0.05).

## Results

### Identification of linked marker(s) with seed size in RIL population using BSA

**Figure 4:**
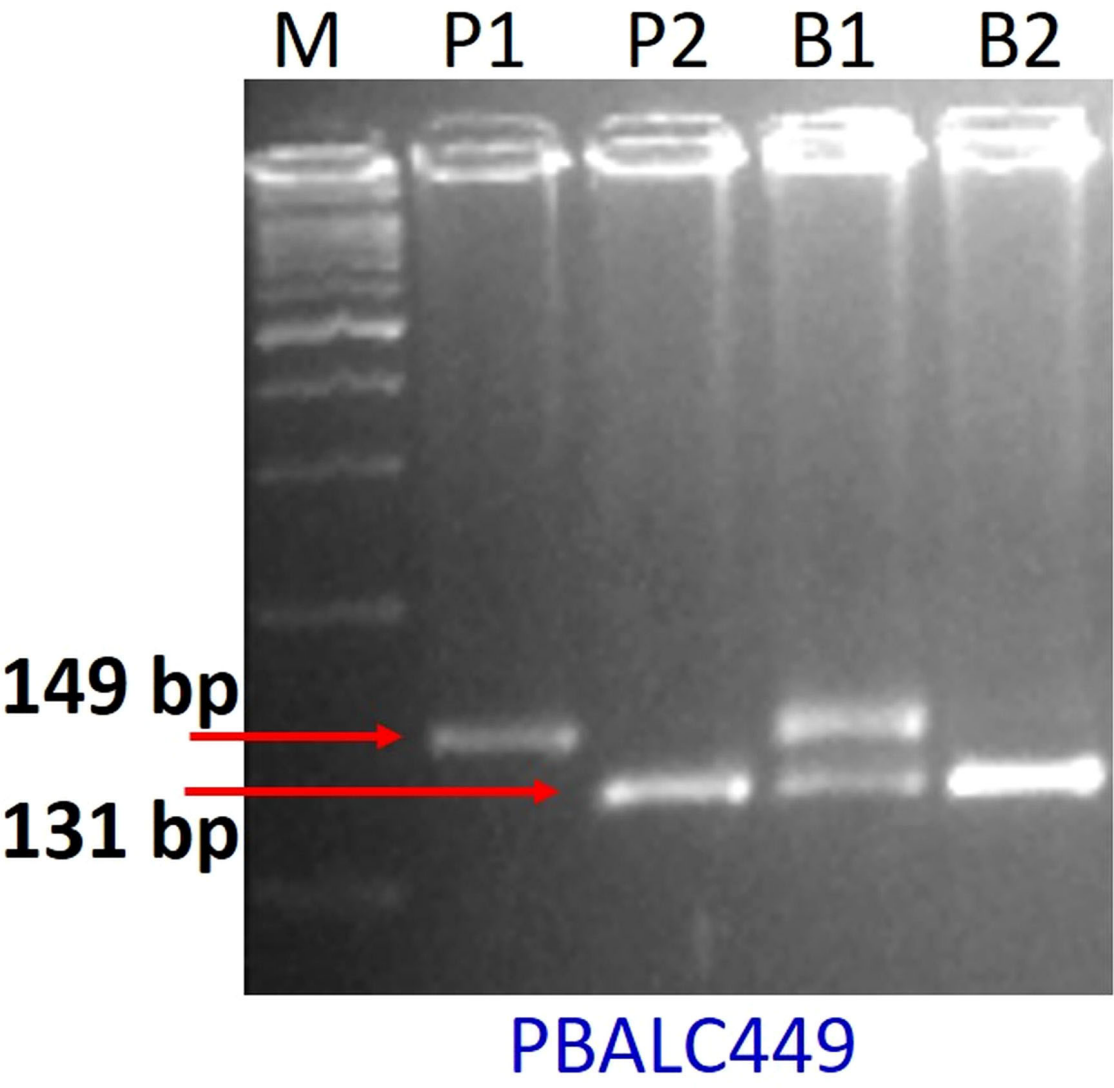
Gel picture showing BSA for seed size with PBALC449 marker. Where, P1: L4602, P2: L830, B1: large seeded bulk, B2: small-seeded bulk, M: DNA ladder.

**Figure 5:**
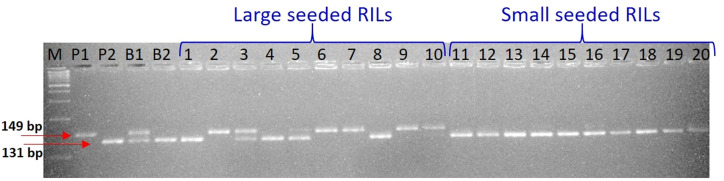
Gel picture showing individual plant analysis (constituting the bulks) for seed size with PBALC449 marker. Where, P1: L4602, P2: L830, B1: Large seeded bulk, B2: Small seeded bulk, 1-10: Individual plants constituting the large-seeded bulk; 11-20: Individual plants constituting the small seeded bulk, M: DNA ladder.

For parental polymorphism, 394 SSR primer pairs of different series like PBALC (Kaur et al., 2011), PLC (Jain et al., 2013), LC (Verma et al., 2014), and GLLC (Saha et al., 2010) have been used, and 31 were found polymorphic (**Tables 1**, **S1**; **Figure S2**). The bulked segregant analysis (BSA) was performed on the parents and the two bulks made by mixing equal quantity of DNA from the 10 extreme RIL genotypes for seed size using polymorphic markers (**Figure 3**). Of 31 polymorphic SSRs, only one PBLAC449, clearly differentiated the small seed size bulk and the parent, whereas large seed size bulk showed two bands. However, other polymorphic markers could not differentiate the bulk (**Figure 4**). The PBLAC449 primer amplified 131bp band for L830 and 149bp band for L4602 genotype. To understand this unique type of banding pattern, the individual plants constituting the bulk was amplified. As observed for the bulked samples, all the 10 plants of small seed size samples showed a band similar to the small seed parent i.e. L830 (131 bp). However, the individual plants constituting the large seed size bulk, a mix of amplification patterns with 03 recombinants (having L830 band size) and 02 heterozygotes were recorded (**Figure 5**).

**Table 1:** Details of the SSRs found polymorphic between the parents L4602 and L830.

S. No	Primer name	Forward sequence (5’-3’)	Reverse Sequence (5’-3’)	Product size (bp)	Motif	Reference
1.	PBALC 114	CACCATAGTGACTACCACCAC	GACAGTGAGGTTGTTGAAAAG	151	(ACC)4	Kaur et al., 2011
2.	PBALC 209	GGAGTTGGTTAGAAGGAAAGA	CTAGATATCATCGATCCATCC	152	(GTC)4	Kaur et al., 2011
3.	PBALC 449	CAGCAATGGTTTTACACTCTC	GGATTTGTTTTGGTTAAGGAT	149	AAC	Kaur et al., 2011
4.	PBALC 761	GTTTGTTATCGTTGGAAGGTT	GAAGCTTAGTGAGAGCAAAAGT	156	CTT	Kaur et al., 2011
5.	PLC 34	TACTGGATGAGACGAAGATGGA	CGAAACCTGGCCTATACAAAAG	190	(T)10	Jain et al., 2013
6.	PLC 36	ACTCAAGTCAACCTCAGAAGGC	CTTAGGAGCCGGAGAAGAAGAT	500	(CTTCA)3	Jain et al., 2013
7.	PLC 37	CTCTCCAGTCCTTGCTTGATG	ACCAACAAACTTGCCAGACTTC	100	(T)13	Jain et al., 2013
8.	PLC 42	AACCAATCATGGCTTCTGCT	TTTCACCGTCTTTATGAACCA	220	(GA)8	Jain et al., 2013
9.	PLC 44	AAATGGTGCATGTGTACGGT	GGAGAACGCGATCAGTAAGG	110	(GCC)5	Jain et al., 2013
10.	PLC 45	CCTTAGTCACTGTGGTCTGATGA	ACAATGAGAGGCCAGTGCTT	390	(A)12	Jain et al., 2013
11.	PLC 51	CCATGATGAGCCTTGAATGA	TCTTCAATCTCCAGGAACACTTT	120	(GAA)10	Jain et al., 2013
12.	PLC 60	TGCTTGGACCCTAAATTTGC	AAGAAAAGGGCAACCACTGA	190	(TA)6	Jain et al., 2013
13.	PLC 66	ATTTGGAGCAAAGATGCAGG	GGATCGACCTCCAATCAAGA	340	(A)10	Jain et al., 2013
14.	PLC 69	CGCTCTACCAACAGCATAA	GAGGTCTCTTTTGTTCTTCACT	210	(CT)19	Jain et al., 2013
15.	PLC 70	CATCTCTTCGTGGCGTAAT	AGCAAACAACAGCACACATA	250	(GTT)9	Jain et al., 2013
16.	PLC 77	GGAAAGAGCCAAGAAGTTG	ACCCATCCTCATCCTTAAAT	230	(CAATGG)5	Jain et al., 2013
17.	PLC 80	GCTAACAAACAACACCATGA	GCATCTAAGTTCTTCAATCTCC	185	(GAA)10	Jain et al., 2013
18.	PLC 105	CTCCCTCAAAATGCGTTGAT	TCCATTACAAGATACTCTCCATGC	320	(TTTTA)6	Jain et al., 2013
19.	LC 272	CAAGATTCCGCACCAATACG	GTTCGGGGGTAATCCAAACT	250	(CT)4	Verma et al., 2014
20.	LC 301	GCCCTAAGTCACCAGAAAACA	CCCTTCGAACCATAATCGTG	300	(GA)4…(GA)5	Verma et al., 2014
21.	LC 305	ACTATTAGCGAAGCCCAGCA	TGAATCCAGAGCCTTTCTTTG	400	CT	Verma et al., 2014
22.	LC 307	AAGTCGACCTTATGAATGAGCA	CAGAACACTGCGAGGTATGA	471	(GA)8…(GA)9	Verma et al., 2014
23.	LC 385	GCCTTTTCAACAGCTACTTTGTT	TGCTTGAGAAATCTGACACACA	392	(CT)7….(GAA)4	Verma et al., 2014
24.	LC 389	TGTCAGCGTAAGATTGGACA	GCAAAGATTTGCTTCAACAAG	384	(CAA)3…(CT)8	Verma et al., 2014
25.	LC 396	GGTCTCTCAAGACTATTGCAAGAAA	TGGATCAAGTGGTATATTTGGACA	292	(AA)3…(GA)10.(CTTT)2	Verma et al., 2014
26.	LC 398	TTGTGGTCACTCAAGACTATTGC	CAAGACTACTCTAGCCTTTTCAACG	392	(CTT)4…(GA)13	Verma et al., 2014
27.	LC 421	CTTTCTTTGAATATGAACGTGAGAG	GCCTTTTCAACGGCTCCT	250	(GA)8	Verma et al., 2014
28.	GLLC 541	TGGGCTCATTGAACCAAAAG	CCCCCTTTTAAGTGATTTTCC	450	–	Saha et al., 2010
29.	GLLC 562	TGTGTAGGCACATCAACAAAA	GGTGGGCATGAGAGGTGTTA	420	–	Saha et al., 2010
30.	GLLC 563	ATGGGCTCATTGAACAAAAG	CCCCCTCTAAGAGATTTTCCTC	300	–	Saha et al., 2010
31.	GLLC 614	AACCCCAGCCAGATCTTACA	AAGGGTGGTTTTGGTCCTATG	–	–	Saha et al., 2010

Afterward, DNA of RILs were rearranged in the order of increasing seed weight, and then PCR (and gel electrophoresis) was done using PBALC449 primer. Of 188 RILs, 39 lines showed 149 bp amplification (as L4602 type), 43 lines showed heterozygous (both 149 and 131 bp bands), and 106 lines showed 131 bp amplification (as L830 type) (**Figure S3**, **Table S2**). Based on the seed size, the RILs were broadly grouped into two categories (i) >24.0 g/1000 seeds (large seeded; 95 Numbers) and (ii) <24.0g/1000 seeds (small seeded; 93 Numbers), expecting that the major locus must have been fixed in a 1:1 ratio in the RILs. Of 188 RILs, the first 73 RILs (15.0 to 21.4 g/1000 seed) which were arranged in the increasing order of seed weight, showed only 03 recombinants (and 07 heterozygotes), while the first 93 lines showed only six recombinants (and 13 heterozygotes). This kind of unique banding pattern has clearly suggested the presence of very tight linkage between small seed size trait and PBLAC449 marker and also indicated that there is no marker distortion in the studied population (**Table 2**).

**Table 2:** The details of the seed size and the amplification pattern of the PBALC449 marker in the 188 RILs.

Band type	Band size (bp)	≤19.7g/1000 seed	>19.7-21g/1000 seed	21-23g/1000 seed	24-30g/1000 seed	>30g/1000 seed	Total
P1 (L4602, Large Seeded)	149	1	2	3	22	11	39
H (Heterozygous)	149 and 131	3	2	8	15	15	43
P2 (L830; Small Seeded)	131	33	25	16	25	7	106
Total	–	37	29	27	62	33	188
		93 (Small Seeded; 06 recombinant and 13 heterozygous)			95 (Large seeded; 32 recombinants and 30 heterozygous)		

Interestingly, this marker showed independent segregation for the large-seeded trait. Thus, it seems that the large seed size expression is being governed by two or more major loci. Since the banding pattern was so unique that we were unable to use any standard marker linkage analysis. To decipher such a unique type of banding pattern, we decided to find the chromosomal location of the amplified product (tightly linked with the small seed size trait only) by cloning, sequencing, and the comparative genomics approach.

### Cloning, sequencing and chromosomal location of PCR amplified products from PBALC449 marker

**Figure 6:**
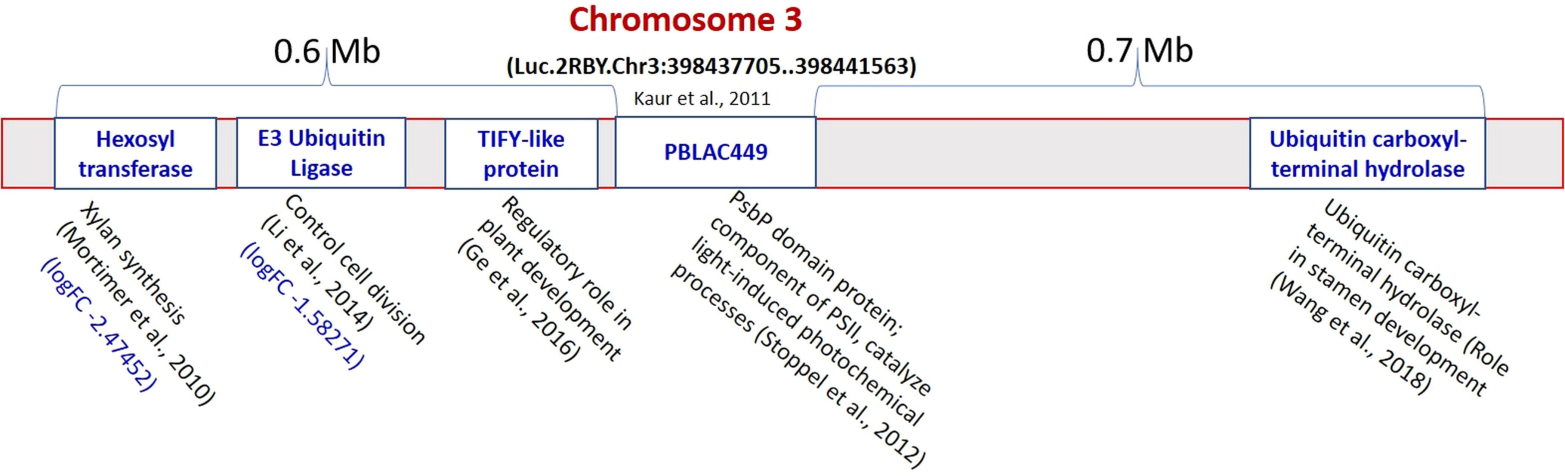
Putative genes identified regulating seed size on both sides (1.2 Mb region) of the PBALC449 in the lentil genome. (Derived from Mortimer et al., 2010; Stoppel et al., 2012; Li and Li, 2014; Ge et al., 2016; Wang et al., 2018).

The pJET1.2 vector was used for cloning of the PCR amplified products (149bp from L4602 and 131bp from L830) from a putatively linked marker viz., PBALC449 for small seed size trait in lentil. The cloned fragment was then sequenced which was further aligned to the reference genome (CDC Redberry Genome Assembly v2.0) using NCBI BLAST browser. The difference in the total length of the amplified product between both parents was due to the presence of 18bp deletion at two places (**Table S3**). The alignment details of the amplified product with the reference genome is presented in **Figures S4**-**S5**. The position of PBALC449 amplification was at Luc.2RBY.Chr3:398437705.398441563 (+strand) which is a PsbP domain protein-encoding gene (3859 bp) and is present on chromosome number 3 of lentil (**Figures 6**, **S6**). The related species sequence similarity showed maximum similarity with *Medicago truncatula* and was followed by *Cicer arietinum* (**Figure S7**). To identify the candidate genes regulating small seed size trait near this marker, we checked RNA Seq data generated by us using the same parental combinations (Dutta et al., 2022) and the physical chromosomal details available at CDC Redberry Genome Assembly v2.0 (Ramsay et al., 2021). Using KnowPulse browser, on the left side (0.6 Mb region) of the PBALC449 amplified region, three candidate genes namely, E3 Ubiquitin ligase (log2FC -1.582), TIFY-like protein, and hexosyltransferase gene (log2FC -2.474); while on the right side (in 0.7 Mb region), a ubiquitin carboxyl-terminal hydrolase gene was found (Ramsay et al., 2021).

### Estimation of lignin, cellulose, xylose, and acetyl content in the parents and the 10 extreme RILs differing for the seed size

Cell wall composition is known to determine the size and shape of some seeds. To validate this, we analyzed and compared the cell wall composition in the mature seeds of parents (L4602, and L830), 10 extreme large-seeded RILs (No. 39, 86, 87, 97, 102, 107, 108, 115, 133, 190) and 10 extreme small-seeded RILs (No. 5, 14, 16, 64, 88, 117, 155, 160, 168, 169) (**Table 3**). Nearly similar FT-IR cellulose content was recorded in the parental genotypes viz., L4602 (24.07%) and L830 (25.96%), while in large-seeded RILs, FT-IR cellulose content was recorded relatively less (21.25 to 28.63%) than that of the small-seeded RILs (22.42 to 39.16%). Lignin is a phenolic polymer that gives rigidity to cell wall, and FT-IR lignin content was recorded more in the small-seeded parental genotype L830 (12.80%) than the large-seeded parental genotype L4602 (11.16%). Similar observations were also recorded for the small-seeded RILs which showed relatively more lignin content (10.73 to 26.85%) than the large-seeded RILs (11.16 to 15.4%). Acetyl bromide soluble lignin (ABSL) content was recorded more in the small-seeded parental genotype L830 (25.07%) than the large-seeded parental genotype L4602 (21.98%). Similarly, small-seeded RILs showed more ABSL content (1.256 to 4.546%) than the large-seeded RILs (1.082 to 2.07%).

**Table 3:** Estimation of cellulose, lignin, acetyl bromide soluble lignin content (ABSL), D- xylose, and acetylated xylose in the mature lentil seeds of 20 RILs (including both parents) and multiple comparison test using Tukey HSD method.

Parameters (Mean ± SD)	FT-IR cellulose (%)	FT-IR lignin (%)	Xylose content (mg/g)	O-Acetyl content (mg/g)	ABSL lignin content (%)
Large seeded					
L4602	24.07 ± 0.714bcdef	11.16 ± 0.049hi	4.163 ± 0.089efghi	**2.013 ± 0.166e**	2.197 ± 0.579cde
RIL 102	21.7 ± 2.263ef	12.99 ± 0.332defgi	**2.219 ± 0.170i**	5.425 ± 0.149bcd	2.070 ± 0.061de
RIL 097	26.1 ± 1.336bcdef	14.63± 0.183def	3.703 ± 0.578fghi	3.696 ± 0.513cde	1.898 ± 0.265de
RIL 039	28.23 ± 0.834bcd	15.15 ± 0.071de	5.401 ± 0.415efghi	3.912 ± 0.273bcde	1.873 ± 0.237de
RIL 107	23.19 ± 0.77cdef	12.15 ± 0.071fghi	3.518 ± 0.108ghi	3.292 ± 0.649de	1.082 ± 0.269e
RIL 190	28.2 ± 0.134bcd	14.63 ± 0.148def	6.492 ± 0.617defgh	5.074 ± 0.363bcd	1.423 ± 0.060e
RIL 133	28.5 ± 1.499bc	14.79 ± 0.82def	6.301 ± 0.634defghi	5.205 ± 0.108bcd	1.425 ± 0.018e
RIL 087	24.8 ± 0.424bcdef	13.75 ± 0.212defgh	6.622 ± 0.362cdefgh	4.290 ± 0.150bcde	1.516 ± 0.639e
RIL 108	28.63 ± 0.395bc	14.37 ± 0.198defg	6.618 ± 0.059cdefgh	5.055 ± 0.431bcd	1.461 ± 0.050e
RIL 086	26.81 ± 0.692bcdef	14.27 ± 0.099defg	7.152 ± 0.267cdefgh	6.138 ± 0.366bc	1.492 ± 0.171e
RIL 115	**21.25 ± 1.018bcd**	15.4 ± 0.989d	4.442 ± 0.053efghi	4.235 ± 0.389bcde	1.879 ± 0.308de
Small seeded					
L830	25.96 ± 0.064bcdef	12.8 ± 0.141defghi	6.86 ± 1.59cdefgh	4.02 ± 1.77bcde	2.506 ± 0.599bcde
RIL 014	29.93 ± 5.487b	13.28 ± 2.347defghi	7.70 ± 2.09bcdef	5.631 ± 0.0158 bcd	1.749 ± 0.300de
RIL 005	25.54 ± 0.346bcdef	11.35 ± 0.1767hi	9.598 ± 0.511abcd	6.514 ± 0.336b	1.456 ± 0.014e
RIL 064	27.68 ± 0.169bcde	12.28 ± 0.1697efghi	3.755 ± 0.376fghi	4.242 ± 0.190bcde	**1.256 ± 0.170e**
RIL 155	28.28 ± 0.049bcd	12.73 ± 0.587defghi	3.084 ± 0.273hi	4.067 ± 0.513bcde	2.475 ± 0.098bcde
RIL 160	25.59 ± 0.078bcdef	11.64 ± 0.318ghi	8.08 ± 1.52abcde	5.402 ± 0.359bcd	1.603 ± 0.250de
RIL 016	22.42 ± 1.407def	**10.73 ± 0.191i**	6.694 ± 0.980cdefgh	5.773 ± 0.344bcd	1.685 ± 0.066de
RIL 088	36.08 ± 0.898a	18.29 ± 0.035c	**12.180 ± 0.120a**	**10.232 ± 0.176a**	3.261 ± 0.692abcd
RIL 168	29.92 ± 0.7b	22.82 ± 0.863b	10.63 ± 2.13abc	6.324 ± 0.872bc	3.948 ± 0.904ab
RIL 169	29.44 ± 1.598b	**26.85 ± 0.629a**	7.19 ± 2.33cdefg	4.831 ± 0.367bcd	**4.546 ± 0.441a**
RIL 117	**39.16 ± 0.382a**	22.27 ± 1.421b	11.568 ± 0.544ab	9.80 ± 1.920a	3.835 ± 0.767abc

Values represent mean ± SD at P ≤ 0.05 and the same lower-case letters within a column are not significantly different. The values in bold represent the higher and lower values.

The xylan was recorded less than the cellulose or lignin in the seeds of parental genotypes and was found more in the small-seeded genotype L830 (6.86 mg/g) than the large-seeded genotype L4602 (4.16 mg/g). Similarly, in large-seeded RILs, xylan content ranged from 2.219 to 7.152 mg/g while in small-seeded RILs, it varied from 3.08 to 12.18 mg/g. Acetyl content was recorded more in small seeded genotype L830 (4.02 mg/g) than that of the large seeded genotype L4602 (2.013 mg/g). Similarly, in large-seeded RILs, acetyl content ranged from 2.013 to 6.138 mg/g; while in small-seeded RILs, it varied from 4.02 to 10.232 mg/g (**Table 3**). In general, cellulose was recorded as the most abundant cell wall component in lentil seeds. Overall, a higher value for almost all the studied cell wall components was recorded for the small-seeded genotype L830 over the large-seeded genotype L4602.

### Characterization of parental genotypes and the RILs using VideometerLab 4.0 for various seed parameters

The lentil parental genotypes L4602 (42.13 g/1000 seeds), L830 (20.90 g/1000 seeds) and the 10 extreme RILs (large seeded RILs: 34.7–39.2 g/1000 seed, and small-seeded RILs: 16.16–20.1 g/1000 seeds) differed significantly for the mean 1000 seed weight, were used for the study. Various other seed parameters like area, length, width, width/length, compactness, width/area, volume, and perimeter were also measured using VideometerLab 4.0 instrument, which showed significant variations for the studied genotypes (**Table 4**). Image of the lentil genotypes (L830 and L4602) as captured by VideometerLab 4.0 at 19 different wavelengths (375 to 970 nm) for further seed parameter analysis is given in **Figure S8**. Interestingly, the mean seed area (mm^2^), length (mm), width (mm), and perimeter (mm) of the parental genotypes L4602 (22.59, 5.57, 5.24, 15.47 respectively) and L830 (11.02, 3.82, 3.71, 10.66 respectively) were found significantly different between these genotypes (**Table 4**). In addition, the 10 RILs (each extreme for seed size) were also compared through one-way ANOVA and were grouped using the Tukey HSD method (p ≤ 0.05). For large seeded RILs, the studied seed parameters (like area: 17.36–20.82 mm^2^, length: 4.9–5.3 mm, width: 4.56–5.05 mm, perimeter: 13.47–14.75 mm) were found significantly higher than the small seeded RILs (area: 8.88–11.03 mm^2^, length: 3.51–3.83 mm, width: 3.28–3.71 mm, perimeter: 9.78–10.7 mm). ANOVA was also performed for all the 188 RILs (including parents) and details are presented in **Table S4**. A representative image (**Figure S7**) shows the details of four large and four small-seeded lentil RIL genotypes as captured by VideometerLab 4.0 at two wavelengths (590 and 850 nm).

**Table 4:** List of seed parameters studied using VideometerLab 4.0 for parents (L4602 and L830) and the 10 extreme RILs. .

Parameters (Mean ± SD)									
Genotypes	1000 seed wt (g)	Area (mm^2^)	Length (mm)	Width (mm)	Width/ length	Compactness (Circle)	Width/ area	Volume (mm^3^)	Perimeter (mm)
Parents									
**L4602**	42.13 ± 1.21a	22.59 ± 1.17a	5.57 ± 0.169a	5.24 ± 0.179a	0.939 ± 0.027cde	0.938 ± 0.025bcd	0.232 ± 0.006j	0.007 ± 0.0002a	15.47 ± 0.452a
**L830**	20.90 ± 1.82d	11.02 ± 0.68g	3.82 ± 0.121gh	3.71 ± 0.117g	0.972 ± 0.015a	0.969 ± 0.014a	0.338 ± 0.011e	0.005 ± 0.0001gh	10.66 ± 0.369h
Large seeded RILs (10 No)									
**RIL039**	**39.20** **± 1.368ab**	20.24 ± 0.633bc	5.24 ± 0.08b	4.94 ± 0.123bcd	**0.942** **± 0.019bcde**	0.941 ± 0.016bcd	0.244 ± 0.003i	0.0065 ± 0.0001b	14.60 ± 0.23bc
**RIL086**	37.43 ± 2.71bc	**17.36** **± 1.396f**	**4.90** **± 0.156f**	**4.56** **± 0.199f**	0.961 ± 0.022e	**0.927** **± 0.037d**	**0.340** **± 0.009f**	**0.00616** **± 0.00019f**	**13.47** **± 0.463g**
**RIL087**	**34.70** **± 0.608c**	18.30 ± 0.889e	4.95 ± 0.119ef	4.77 ± 0.148e	0.964 ± 0.019abc	0.961 ± 0.017ab	0.261 ± 0.006fg	0.006223 ± 0.00015ef	13.77 ± 0.332fg
**RIL097**	37.70 ± 0.608bc	**20.82** **± 0.737b**	**5.30** **± 0.114b**	**5.05** **± 0.117b**	0.952 ± 0.025abcde	0.952 ± 0.022abcd	**0.242** **± 0.006i**	**0.006668** **± 0.00014b**	**14.75** **± 0.266b**
**RIL102**	39.16 ± 1.607ab	18.73 ± 0.614e	5.06 ± 0.113cde	4.85 ± 0.147de	0.958 ± 0.035abcd	0.955 ± 0.028abc	0.259 ± 0.007fgh	0.006366 ± 0.00014cde	14.27 ± 0.391cde
**RIL107**	38.46 ± 0.924abc	19.57 ± 0.74cd	5.17 ± 0.124bc	4.9 ± 0.116cde	0.948 ± 0.022abcde	0.946 ± 0.023abcd	0.250 ± 0.005ghi	0.006507 ± 0.00016bc	14.27 ± 0.25cde
**RIL108**	35.93 ± 1.102bc	20.09 ± 0.658bc	5.19 ± 0.123bc	5.03 ± 0.096bc	**0.968** **± 0.016ab**	**0.968** **± 0.014a**	0.250 ± 0.005hi	0.006527 ± 0.00015bc	14.43 ± 0.272bcd
**RIL115**	35.73 ± 1.419bc	18.92 ± 0.902de	5.09 ± 0.117cd	4.83 ± 0.169de	0.949 ± 0.029abcde	0.947 ± 0.028abcd	0.255 ± 0.006fgh	0.006396 ± 0.00015cd	14.21 ± 0.299de
**RIL133**	35.56 ± 0.513bc	20.63 ± 0.608b	5.28 ± 0.086b	5.04 ± 0.088bc	0.954 ± 0.018abcde	0.953 ± 0.018abcd	0.244 ± 0.004i	0.006643 ± 0.00011bde	14.67 ± 0.269b
**RIL190**	36.43 ± 0.512bc	18.83 ± 0.794de	5.05 ± 0.121de	4.83 ± 0.104de	0.957 ± 0.015abcde	0.959 ± 0.016ab	0.256 ± 0.006fgh	0.006344 ± 0.00015de	13.98 ± 0.349ef
Small seeded RILs (10 No)									
**RIL005**	**20.1** **± 1.85de**	**11.03** **± 0.462 g**	**3.83** **± 0.076g**	**3.71** **± 0.112g**	**0.967** **± 0.016abc**	0.968 ± 0.014a	**0.336** **± 0.0062e**	**0.00482** **± 0.000096g**	**10.7** **± 0.23h**
**RIL014**	18.13 ± 1.026de	10.57 ± 0.466gh	3.79 ± 0.09ghi	3.60 ± 0.089gh	0.947 ± 0.026abcde	0.948 ± 0.027abcd	0.341 ± 0.0113de	0.004768 ± 0.000113ghi	10.4 ± 0.224hi
**RIL016**	17.03 ± 1.002de	10.67 ± 0.438gh	3.79 ± 0.088ghi	3.64 ± 0.098gh	0.960 ± 0.023abcd	0.960 ± 0.023ab	0.341 ± 0.0085de	0.004763 ± 0.000112ghi	10.52 ± 0.222hi
**RIL064**	17.83 ± 1.041de	9.57 ± 0.309ij	3.60 ± 0.079Jkl	3.42 ± 0.076ij	0.950 ± 0.027abcde	0.952 ± 0.022abcd	0.357 ± 0.0097bc	0.004526 ± 0.0001jkl	10.01 ± 0.184jkl
**RIL088**	17.3 ± 0.608de	**8.88** **± 0.481j**	**3.51** **± 0.095l**	**3.28** **± 0.113k**	**0.934** **± 0.031de**	**0.93** **± 0.033cd**	**0.369** **± 0.0128a**	**0.004413** **± 0.000121l**	9.79 ± 0.319l
**RIL111**	18.6 ± 1.637de	9.16 ± 0.355j	3.55 ± 0.085kl	3.36 ± 0.079jk	0.940 ± 0.029abcde	0.938 ± 0.028bcd	0.367 ± 0.011ab	0.004469 ± 0.000107kl	9.89 ± 0.263kl
**RIL117**	16.66 ± 1.528e	9.23 ± 0.483j	3.54 ± 0.124kl	3.37 ± 0.073jk	0.951 ± 0.028abcde	0.951 ± 0.023abcd	0.365 ± 0.0137ab	0.004456 ± 0.000156kl	**9.78** **± 0.292l**
**RIL155**	17.13 ± 1.026de	10.09 ± 0.768hi	3.66 ± 0.129ijk	3.55 ± 0.146hi	0.9672 ± 0.021ab	**0.97** **± 0.017a**	0.352 ± 0.0146c	0.004609 ± 0.000162ijk	10.23 ± 0.405ijk
**RIL160**	18.8 ± 1.311de	10.13 ± 0.449hi	3.69 ± 0.085hij	3.56 ± 0.083hi	0.963 ± 0.015abc	0.962 ± 0.015ab	0.351 ± 0.008c	0.004647 ± 0.000108hij	10.23 ± 0.259ijk
**RIL169**	**16.16** **± 0.289e**	10.3 ± 0.431ghi	3.73 ± 0.079ghi	3.58 ± 0.072gh	0.960 ± 0.014abcd	0.962 ± 0.015ab	0.348 ± 0.0091cd	0.004692 ± 0.0001ghi	10.35 ± 0.225hij

### Values represent mean ± SD at P ≤ 0.05. Same lower-case letters within a column are not significantly different. The values in bold represent the higher and lower values.Validation of identified candidate genes in a RIL mapping population

Another mapping population (RIL; F_3:4_) which was derived from the cross between Globe mutant (1000 seed weight=13.6g) and L4775 (1000 seed weight=28.47g) was used for the validation. Two contrasting bulks using 20 extreme plants each for the seed weight (small seed bulk: 1000 seed weight=18.57g; bold seed bulk: 1000 seed weight=24.46g) along with a parent (Globe Mutant) was used for the whole genome resequencing (WGRS). Detailed sequence analysis could identify 90 SNPs/InDels for the four candidate genes as identified by the BLAC449 marker (**Table S5**).

For *E3 ubiqutin ligase* gene 03 SNPs was identified; whereas for *TIFY-like protein* gene, 34 SNPs and 01 InDel was identified and most of these showed modifier effect. Among the 20 SNPs and 02 InDels of *Hexosyltransferase* gene, one InDel showed disruptive inframe deletion with moderate effect while other SNPs showed mostly missense variant with moderate effect. Similarly, for the *Ubiquitin carboxyl-terminal hydrolase* gene we have identified 30 SNPs and most of these showed modifier effect (**Table S5**).

## Discussion

### BSA and identification of candidate genes regulating seed size trait in lentil

A total of 394 SSR diverse SSR primer pairs (Saha et al., 2010; Kaur et al., 2011; Jain et al., 2013; Verma et al., 2014) were used and 31 were found polymorphic, which is 7.9% of the total primers used. A similar level of polymorphism was also reported by previous workers (Kumar et al., 2019; Singh et al., 2019). Of all the polymorphic SSRs, only one (PBLAC449) could differentiate the small seed size bulk and the parent, while the large seed size bulk showed two bands. This kind of unique polymorphism pattern was not yet reported in the lentil. Detailed RIL analysis (188 No) using PBLAC449 marker showed that the region near the PBLAC449 marker, seems to regulate the small seed size trail while large seed size is being governed by more than one locus. Moreover, quantitative regulation of seed size trait is reported by a number of workers (Fedoruk et al., 2013; Verma et al., 2015; Khazaei et al., 2018).

To understand this unique type of banding pattern, and to find the chromosomal location of amplified product (tightly linked with the small seed size trait only); cloning, sequencing, and the comparative genomics approaches were used. The PCR amplified fragment was cloned, sequenced, and aligned to the recently released lentil reference genome (CDC Redberry Genome Assembly v2.0) (Ramsay et al., 2021). The PCR amplified product from PBALC449 got aligned at Luc.2RBY.Chr3:398437705.398441563 (+strand) on chromosome number 3 and is a PsbP domain protein-encoding gene (3859 bp) (**Figure S5**). Similarly, Verma et al. (2015) have identified three major QTLs for seed weight and seed size traits in lentils on LG4; while, Fedoruk et al. (2013) have identified three QTLs for seed diameter on LG1, 2, and 7 which together explained >60% of the PVE and Khazaei et al. (2018) have identified two associated SNPs with seed diameter (*viz*. LcC09638p190 and LcC08798p992) on chromosomes 1 and 7, respectively. In addition, QTLs for seed weight (Abbo et al., 1991) and seed diameter (Fratini et al., 2007) are identified in lentils.

Further, to identify the candidate genes regulating small seed size trait near this marker (1.4 Mb region), we analyzed our RNA Seq data (Dutta et al., 2022). On the left side of the PBALC449 amplified region (0.6 Mb), three genes namely, E3 ubiquitin ligase (log2FC -1.582), hexosyltransferase (log2FC -2.474), and TIFY-like protein gene were found, while on the right side (0.7 Mb) a ubiquitin carboxyl-terminal hydrolase gene was found (**Figure 6**). The E3 Ubiquitin ligase gene is known to have a role in controlling cell division (Li and Li, 2014); while the TIFY-like protein gene is having a role in regulating the process of plant development (Ge et al., 2016). Similarly, the hexosyltransferase gene is known to have a role in the regulation of xylan synthesis (Mortimer et al., 2010); while ubiquitin carboxyl-terminal hydrolase gene is required for periodic maintenance of the circadian clock (Hayama et al., 2019) and inflorescence architecture (Yang et al., 2007) in *Arabidopsis*.

Domoney et al. (2006) reported two distinct phases during seed development in the legumes. In the first phase, cell division (in seeds) is dependent on embryo genotype having certain loci controlling the cotyledon cell number and is largely insensitive to environmental cues. Thus, this phase mainly controls the seed diameter and seed plumpness. The second phase regulates seed thickness *via* cell expansion, which is highly influenced by the environment and is mainly regulated by photosynthate partitioning loci. The seed size is reportedly influenced by both pre-anthesis and post-anthesis periods (Gupta et al., 2006) as these affect the amount of assimilates partitioned to the developing seeds (Pre-anthesis) and also the time for seed maturation (post-anthesis) which could alter the seed size. Flowering time and other flower morphology-related loci were also known to control the seed size in model legume crops (Ohto et al., 2005; He et al., 2010; Wang et al., 2012). In chickpea, a major flowering time gene, PPD, is reported to affect the seed weight, and early flowering results in reduced seed weight (Hovav et al., 2003). Validation results in another mapping population (Globe mutant × L4775) using WGRS also confirmed the presence of SNPs and InDels in the four candidate genes. However, there is still a need to validate these candidate genes having a role in the seed size regulation, in different lentil genotypes for its ultimate application in the breeding program aiming for seed size improvement.

### Seed biochemical parameters

Seed size and shape are regulated by the cell wall composition in lentils (Dutta et al., 2022). However, no other detailed report mentioning the relationship between the seed size and cell wall composition including cellulose, lignin, and xylose in lentils is known. The data of parents and the 10 extreme RILs for the cell wall composition in the mature seeds showed significant variations for parameters like FT-IR cellulose, FT-IR lignin, ABSL, xylan, and acetyl content (**Table 3**). In small seeded RILs, in general, more of FT-IR cellulose (22.4 to 39.16%), FT-IR lignin (10.73 to 26.85%), ABSL (1.26 to 4.55%), xylose content (3.083 to 12.18 mg/g), acetyl content (4.02 to 10.23 mg/g) was recorded than the large-seeded RILs (FT-IR cellulose: 21.25 to 28.6%; FT-IR lignin: 11.16 to 15.4%; ABSL: 1.08 to 2.19%; xylose content: 2.22 to 7.15 mg/g; acetyl content: 2.01 to 6.14 mg/g). Overall, cellulose was recorded as the most abundant cell wall component in lentil seeds. Similarly, cellulose and hemicellulose such as galactomannan, mannan, and xyloglucan were found to play a crucial function in determining the shape and size of both developing and mature seeds (Buckeridge, 2010).

This study recorded up to 39.16% cellulose (FT-IR) in lentil seeds, whereas in different plant species nearly 40–60% cellulose was reported (Costa and Plazanet, 2016). In the RILs, 10.73 to 26.85% lignin (FT-IR) was recorded whereas 5.13% mean lignin content was recorded in soybean seeds (Krzyzanowski et al., 2008), and genotypes having >5% lignin in the seed coat were less prone to mechanical damage (Alvarez et al., 1997). The presence of more lignin in lentil seeds over soybean may be due to the presence of more colored compounds in the lentil seed coat (Dutta et al., 2022). Xylose and xyloglucan are considered important seed storage polysaccharides, especially in developing seeds (Buckeridge, 2010). The studied lentil genotypes showed 2.22 to 12.18 mg/g xylose content, whereas 3.5–4.5% (dry weight basis) acetyl–xylose content was recorded in the hardwoods (Teleman et al., 2002). Acetyl content in the range of 2.01 to 10.23 mg/g was recorded in the studied RILs. Differential seed sizes in different genotypes might be due to the different levels of polysaccharides acetylation which seems to affect their water solubility, interactions with cellulose, and various other physicochemical properties (Busse-Wicher et al., 2014).

RNA-seq results of Dutta et al. (2022) identified various cell wall-associated GO terms and also the differential expression of xyloglucan endotransglucosylase encoding gene, suggesting their involvement in the cell wall synthesis during seed development in lentils, and similar results were also recorded in soybean (Du et al., 2017). Overall, a higher value for almost all the studied cell wall components for small-seeded lentil genotype (L830) over large-seeded (L4602) genotype needs further detailed stage-specific investigations.

### Characterization of lentil genotypes using VideometerLab 4.0 for various seed parameters

In general, seed size in lentils is mostly determined using a very crude method of measuring 100 or 1000 seed weight (Tullu et al., 2001). Even in soybean, seed shape parameters are measured using a caliper (Xu et al., 2011), while in chickpeas Hossain et al. (2010) used seed sizing using graded sieves for determining the seed size and shape. By this, it is impossible to determine the seed thickness or seed plumpness (Dutta et al., 2022). However, in this study, VideometerLab 4.0 instrument was used to measure various seed parameters like area, length, width, width/length, compactness, width/area, volume, and perimeter of all the RILs (188 No) and its parents. Most of the studied parameters showed significant variations for the studied genotypes (**Table S4**). For large seeded RILs and parents, 1000 seed weight (34.7 to 42.13 g), area (17.36 to 22.59 mm^2^), seed length (4.9 to 5.57 mm), seed width (4.56 to 5.24 mm), and seed perimeter (13.47 to 15.47 mm) were found significantly more than the small seeded RILs including parent (1000 seed weight: 16.16 to 20.90 g, area: 8.88 to 11.03 mm^2^, seed length: 3.51 to 3.83 mm, seed width: 3.28 to 3.71 mm, and seed perimeter: 9.78 to 10.7 mm). Similarly, Shahin et al. (2012) used cameras and captured the 3-dimensional lentil seed images and measured the seed plumpness; while Shahin and Symons (2001) deployed computer-aided two-dimensional imaging to measure the diameter of lentil seeds. Similarly, previous studies also demonstrated huge variations for various seed parameters in lentils (Tullu et al., 2001; Tripathi et al., 2022). Thus, the use of VideometerLab was found very precise, quick, and easy method for the determination of several seed parameters.

## Conclusions

Results of the study have conclusively shown the importance of the maker PBLAC449 in the identification of genotypes having small seed size in lentils. In addition, the region identified on chromosome 03, needs more critical attention for the validation of genes regulating the seed size trait in lentils. The cell wall composition including cellulose, xylan, etc. was extensively analyzed, using wet chemistry methods and FT-IR to understand the association between cell extensibility and the seed size in lentils. Compared to any other method, the use of VideometerLab 4.0 was found very effective, easy, and quick, and should be used for the measurement of various essential seed parameters in lentils. Thus, the information generated in this study has paved the way for further in-depth analysis of the factors governing seed size in lentils including the development of genotypes having customized seed sizes.

## Data availability statement

The original contributions presented in the study are included in the article/**Supplementary Material**. Further inquiries can be directed to the corresponding authors.

## Author contributions

**Conceptualization**: GM, HKD, SG, and SK; **methodology**: HD, SM, SS, MA, MT, SD, PP, AK, KT, and RK; **formal analysis:** HD, SM, DV, DM, and AS; **resources**: GM, SK, and HKD; **data curation**: HKD and GM; **writing—original draft preparation**: HD, GM, and HKD; **writing—review and editing**: HKD, GM, SG, and SK; **supervision**: GM and HKD. All authors contributed to the article and approved the submitted version.

## Supplementary material

Supplementary Figure 1 Seed size variation among parents (L4602 and L830) and the 10 extreme RILs for large and small seed size.

Supplementary Figure 2 A representative gel pictures showing results of parental polymorphism between L4602 and L830 for various SSR markers. Where 1. is L4602 and 2. is L830

Supplementary Figure 3 BLAST for sequence from large seeded parent (L4602) (149 bp).

Supplementary Figure 4 BLAST for sequence from small seeded parent (L830) (131 bp; due to 18 bp deletion at two places).

Supplementary Figure 5 Identified position of the SSR (PBALC 449) in the lentil genome (Chromosome 3).

Supplementary Figure 6 Sequnce similarity of the marker (PBALC449) with the relative sp. It is showing most similarity to *Medicago truncatula*, followed by *Cicer arietinum*.

Supplementary Figure 7 Representative image of eight lentil RIL genotypes, captured by VideometerLab 4.0 at two wavelengths (590 and 850 nm) for further seed parameter analysis.

Supplementary Figure 8 Image of the lentil genotypes (L830 and L4602) captured by VideometerLab 4.0 at 19 wavelengths (375 to 970 nm) for further seed parameter analysis.

## References

[B1] Abbo S. Ladizinsky G. Weeden N. F. (1991). Genetic-analysis and linkage study of seed weight in lentil. Euphytica 58, 259–266. doi: 10.1007/BF00025258

[B2] Alvarez P. J. C. Krzyzanowski F. C. Mandarino J. M. G. França Neto J. B. (1997). Relationship between soybean seed coat lignin content and resistance to mechanical damage. Seed. Sci. Technol. 25, 209–214.

[B3] Arumuganathan K. Earle E. D. (1991). Nuclear DNA content of some important plant species. Plant Mol. Biol. Rep. 9, 208–218. doi: 10.1007/BF02672069

[B4] Barulina H. (1930). Lentils of the USSR and other countries (English summary). Bull. Appl. Bot. Genet. Plant Breed. 40, 265–304.

[B5] Buckeridge M. S. (2010). Seed cell wall storage polysaccharides: Models to understand cell wall biosynthesis and degradation. Plant Physiol. 154 (3), 1017–1023. doi: 10.1104/pp.110.158642 20855518 PMC2971584

[B6] Busse-Wicher M. Gomes T. C. Tryfona T. Nikolovski N. Stott K. Grantham N. J.. (2014). The pattern of xylan acetylation suggests xylan may interact with cellulose microfibrils as a twofold helical screw in the secondary plant cell wall of *Arabidopsis thaliana* . Plant J. 79 (3), 492–506. doi: 10.1111/tpj.12575 24889696 PMC4140553

[B7] Canteri M. Renard C. Le Bourvellec C. Bureau S. (2019). ATR-FTIR spectroscopy to determine cell wall composition: Application on a large diversity of fruits and vegetables. Carbohydr. Polymers. 212, 186–196. doi: 10.1016/j.carbpol.2019.02.021 30832846

[B8] Costa G. Plazanet I. (2016). Plant cell wall, a challenge for its characterisation. Adv. Biol. Chem. 6, 70–105. doi: 10.4236/abc.2016.63008

[B9] Dikshit H. K. Mishra G. P. Aski M. S. Singh A. Tripathi K. Bansal R.. (2022a). “Lentil breeding,” in Fundamentals of field crop breeding. Eds. Yadava D. K. Dikshit H. K. Mishra G. P. Tripathi S. (Singapore: Springer), 1181–1236. doi: 10.1007/978-981-16-9257-4_24

[B10] Dikshit H. K. Mishra G. P. Aski M. Singh A. Virk P. S. Kumar S. (2022b). “Lentil biofortification,” in Biofortification of staple crops. Eds. Kumar S. Dikshit H. K. Mishra G. P. Singh A. (Singapore: Springer), 271–293. doi: 10.1007/978-981-16-3280-8_11

[B11] Domoney C. Duc G. Ellis T. H. Ferrándiz C. Firnhaber C. Gallardo K.. (2006). Genetic and genomic analysis of legume flowers and seeds. Curr. Opin. Plant Biol. 9, 133–141. doi: 10.1016/j.pbi.2006.01.014 16480914

[B12] Du J. Wang S. He C. Zhou B. Ruan Y. L. Shou H.. (2017). Identification of regulatory networks and hub genes controlling soybean seed set and size using RNA sequencing analysis. J. Exp. Bot. 68 (8), 1955–1972. doi: 10.1093/jxb/erw460 28087653 PMC5429000

[B13] Dutta H. Mishra G. P. Aski M. S. Bosamia T. C. Mishra D. C. Bhati J.. (2022). Comparative transcriptome analysis, unfolding the pathways regulating the seed-size trait in cultivated lentil (*Lens culinaris* medik.). Front. Genet. 13. doi: 10.3389/fgene.2022.942079 PMC9399355 36035144

[B14] Erskine W. Williams P. C. Nakkoul H. (1991). Splitting and dehulling lentil (*Lens culinaris*): Effects of seed size and different pretreatments. J. Sci. Food Ag. 57, 77–84. doi: 10.1002/jsfa.2740570109

[B15] FAOSTAT (2020) Statistical databases (Italy: Food and Agriculture Organization of the United Nations). Available at: https://www.fao.org/faostat/en/#home (Accessed Aug 14, 2022).

[B16] Fedoruk M. Vandenberg A. Bett K. (2013). Quantitative trait loci analysis of seed quality characteristics in lentil using single nucleotide polymorphism markers. Plant Genome 6 (3), 1–10. doi: 10.3835/plantgenome2013.05.0012 PMC12962556 29505642

[B17] Ford R. Rubeena Redden R. J. Materne M. Taylor P. W. J.. (2007). Lenti. In: Kole C. eds. Genome Mapping and Molecular Breeding in Plants (Berlin, Heidelberg: Springer) 3, 91–108. doi: 10.1007/978-3-540-34516-9_5

[B18] Foster C. E. Martin T. M. Pauly M. (2010). Comprehensive compositional analysis of plant cell walls (lignocellulosic biomass) part I: lignin. J. Visualized. Experiments. 37, 1745. doi: 10.3791/1745 PMC3144576 20224547

[B19] Fratini R. Duran Y. Garcia P. Perez de la Vega M. (2007). Identification of quantitative trait loci (QTL) for plant structure, growth habit and yield in lentil. Spanish. J. Ag. Res. 5, 348–356. doi: 10.5424/sjar/2007053-255

[B20] García-Alegría A. Anduro-Corona I. Pérez-Martínez C. Guadalupe Corella-Madueño M. Rascón-Durán M. Astiazaran-Garcia H. (2020). Quantification of DNA through the NanoDrop spectrophotometer: Methodological validation using standard reference material and sprague dawley rat and human DNA. Int. J. Anal. Chem. 2020, 1–9. doi: 10.1155/2020/8896738 PMC7719535 33312204

[B21] Ge L. Yu J. Wang H. Luth D. Bai G. Wang K.. (2016). Increasing seed size and quality by manipulating BIG SEEDS1 in legume species. Proc. Nat. Acad. Sci. U.S.A. 113 (44), 12414–12419. doi: 10.1073/pnas.1611763113 PMC5098654 27791139

[B22] Gupta P. K. Rustgi S. Kumar N.. (2006). Genetic and molecular basis of grain size and grain number and its relevance to grain productivity in higher plants. Genome 49 (6), 565–571. doi: 10.1139/g06-063 16936836

[B23] Hamdi A. Erskine W. Gates P.. (1991). Relationships among economic characters in lentil. Euphytica 57 (2), 109–116. doi: 10.1007/bf00023068

[B24] Hayama R. Yang P. Valverde F. Mizoguchi T. Furutani-Hayama I. Vierstra R. D.. (2019). Ubiquitin carboxyl-terminal hydrolases are required for period maintenance of the circadian clock at high temperature in *Arabidopsis* . Sci. Rep. 9, 17030. doi: 10.1038/s41598-019-53229-8 31745110 PMC6863813

[B25] He C. Tian Y. Saedler R. Efremova N. Riss S. Khan M. R.. (2010). The MADS-domain protein MPF1 of *Physalis floridana* controls plant architecture, seed development and flowering time. Planta 231, 767–777. doi: 10.1007/s00425-009-1087-z 20033229 PMC2806528

[B26] Hossain S. Ford R. McNeil D. Pittock C. Panozzo J. F. (2010). Development of a selection tool for seed shape and QTL analysis of seed shape with other morphological traits for selective breeding in chickpea (*Cicer arietinum* l.). Aus. J. Crop Sci. 4, 278–288.

[B27] Hovav R. Upadhyaya K. C. Beharav A. Abbo S. (2003). Major flowering time gene and polygene effects on chickpea seed weight. Plant Breed. 122, 539–541. doi: 10.1111/j.1439-0523.2003.00895.x

[B28] Jain N. Dikshit H. K. Singh D. Singh A. Kumar H. (2013). Discovery of EST-derived microsatellite primers in the legume *Lens culinaris* (Fabaceae). Appl. Pl. Sci. 1 (7), 1200539. doi: 10.3732/apps.1200539 PMC4103130 25202567

[B29] Kaur S. Cogan N. Pembleton L. Shinozuka M. Savin K. Materne M.. (2011). Transcriptome sequencing of lentil based on second-generation technology permits large-scale unigene assembly and SSR marker discovery. BMC Genomics 12 (1), 265. doi: 10.1186/1471-2164-12-265 21609489 PMC3113791

[B30] Khazaei H. Fedoruk M. Caron C. Vandenberg A. Bett K. (2018). Single nucleotide polymorphism markers associated with seed quality characteristics of cultivated lentil. Plant Genome 11 (1), 170051. doi: 10.3835/plantgenome2017.06.0051 PMC12962556 29505642

[B31] Krzyzanowski F. Franca Neto J. Mandarino J. Kaster M. (2008). Evaluation of lignin content of soybean seed coat stored in a controlled environment. Rev. Bras. Sementes. 30 (2), 220–223. doi: 10.1590/s0101-31222008000200028

[B32] Kumar H. Singh A. Dikshit H. K. Mishra G. P. Aski M. Meena M. C.. (2019). Genetic dissection of grain iron and zinc concentrations in lentil (*Lens culinaris* medik.). J. Genet. 98 (3), 66. doi: 10.1007/s12041-019-1112-3 31544775

[B33] Labbe N. Rials T. Kelley S. Cheng Z. Kim J. Li Y. (2005). FT-IR imaging and pyrolysis-molecular beam mass spectrometry: new tools to investigate wood tissues. Wood Sci. Technol. 39 (1), 61–76. doi: 10.1007/s00226-004-0274-0

[B34] Li N. Li Y. (2014). Ubiquitin-mediated control of seed size in plants. Front. Plant Sci. 5. doi: 10.3389/fpls.2014.00332 PMC4093792 25071811

[B35] Michelmore R. Paran I. Kesseli R. (1991). Identification of markers linked to disease-resistance genes by bulked segregant analysis: a rapid method to detect markers in specific genomic regions by using segregating populations. Proc. Nat. Acad. Sci. U.S.A. 88 (21), 9828–9832. doi: 10.1073/pnas.88.21.9828 PMC52814 1682921

[B36] Mishra G. P. Ankita Aski M. S. Tontang M. T. Priti C. Tripathi K.. (2022b). Morphological, molecular, and biochemical characterization of a unique lentil (*Lens culinaris* medik.) genotype showing seed-coat color anomalies due to altered anthocyanin pathway. Plants 11 (14), 1815. doi: 10.3390/plants11141815 35890449 PMC9319573

[B37] Mishra G. P. Aski M. S. Bosamia T. Chaurasia S. Mishra D. C. Bhati J.. (2022a). Insights into the host-pathogen interaction pathways through RNA-seq analysis of *Lens culinaris* medik. in response to *Rhizoctonia bataticola* infection. Genes 13 (1), 90. doi: 10.3390/genes13010090 PMC8774501 35052429

[B38] Mishra G. P. Dikshit H. K. Kumari J. Priti Tripathi K. Devi J.. (2020). Identification and characterization of novel penta-podded genotypes in the cultivated lentil (*Lens culinaris* medik.). Crop Sci. 60 (4), 1974–1985. doi: 10.1002/csc2.20156

[B39] Mishra G. P. Singh R. K. Mohapatra T. Singh A. K. Prabhu K. V. Zaman F. U. (2001). Molecular mapping of a fertility restorer gene in basmati rice using microsatellite markers. Indian J. Genet. Plant Breed. 61 (4), 348–349.

[B40] Mishra G. P. Singh R. K. Mohapatra T. Singh A. K. Prabhu K. V. Zaman F. U.. (2003). Molecular mapping of a gene for fertility restoration of wild abortive (WA) cytoplasmic male sterility using a basmati line restorer line. J. Plant Biochem. Biotechnol. 12, 37–42. doi: 10.1007/BF03263157

[B41] Mortimer J. Miles G. Brown D. Zhang Z. Segura M. Weimar T.. (2010). Absence of branches from xylan in *Arabidopsis* gux mutants reveals potential for simplification of lignocellulosic biomass. Proc. Nat. Acad. Sci. U.S.A. 107 (40), 17409–17414. doi: 10.1073/pnas.1005456107 PMC2951434 20852069

[B42] Murray M. Thompson W. (1980). Rapid isolation of high molecular weight plant DNA. Nucleic Acids Res. 8 (19), 4321–4326. doi: 10.1093/nar/8.19.4321 7433111 PMC324241

[B43] Ohto M. A. Fischer R. L. Goldberg R. B. Nakamura K. Harada J. J. (2005). Control of seed mass by *APETALA2* . Proc. Nat. Acad. Sci. U.S.A. 102, 3123–3128. doi: 10.1073/pnas.0409858102 PMC549491 15708976

[B44] Pawar P. Derba-Maceluch M. Chong S. Gandla M. Bashar S. Sparrman T.. (2017). In muro deacetylation of xylan affects lignin properties and improves saccharification of aspen wood. Biotechnol. Biofuels 10 (1), 98. doi: 10.1186/s13068-017-0782-4 28428822 PMC5397736

[B45] Priti. Mishra G. P. Dikshit H. K. Vinutha T. Mechiya T. Stobdan T.. (2021). Diversity in phytochemical composition, antioxidant capacities, and nutrient contents among mungbean and lentil microgreens when grown at plain-altitude region (Delhi) and high-altitude region (Leh-ladakh), India. Front. Plant Sci. 12. doi: 10.3389/fpls.2021.710812 PMC8420906 34497624

[B46] Priti. Sangwan S. Kukreja B. Mishra G. P. Dikshit H. K. Singh A.. (2022). Yield optimization, microbial load analysis, and sensory evaluation of mungbean (*Vigna radiata* l.), lentil (*Lens culinaris* subsp. *culinaris*), and Indian mustard (*Brassica juncea* l.) microgreens grown under greenhouse conditions. PloS One 17, e0268085. doi: 10.1371/journal.pone.0268085 35609036 PMC9128967

[B47] Ramsay L. Koh C. S. Kagale S. Gao D. Kaur S. Haile T.. (2021) Genomic rearrangements have consequences for introgression breeding as revealed by genome assemblies of wild and cultivated lentil species. bioRxiv. 2021 jul 24. Available at: https://knowpulse.usask.ca/genome-assembly/Lcu.2RBY.

[B48] Rastogi L. Chaudhari A. Sharma R. Pawar P. (2022). Arabidopsis GELP7 functions as a plasma membrane-localized acetyl xylan esterase, and its overexpression improves saccharification efficiency. Plant Mol. Biol. 109 (6), 781–797. doi: 10.1007/s11103-022-01275-8 35577991

[B49] Saha G. Sarker A. Chen W. Vandemark G. Muehlbauer F. (2010). Inheritance and linkage map positions of genes conferring resistance to *Stemphylium* blight in lentil. Crop Sci. 50 (5), 1831–1839. doi: 10.2135/cropsci2009.12.0709

[B50] Sambrook J. Fritsch E. R. Maniatis T. (1989). Molecular cloning: A laboratory manual. 2nd ed (Cold Spring Harbor, NY: Cold Spring Harbor Laboratory Press), 1546, ISBN: ISBN:9780879693091.

[B51] Sandhu J. Singh S. (2007). History and origin. In: Eds. Yadav S. S. McNeil D. L. Stevenson P. C. Lentil. (Dordrecht: Springer). doi: 10.1007/978-1-4020-6313-8_1

[B52] Shahin M. A. Symons S. J. (2001). A machine vision system for grading lentils. Cand. Biosys. Eng. 43, 7–7.

[B53] Shahin M. A. Symons S. J. Poysa V. W. (2006). Determining soya bean size uniformity using image analysis. Biosys. Eng. 94, 191–198. doi: 10.1016/j.biosystemseng.2006.02.011

[B54] Shahin M. A. Symons S. J. Wang N. (2012). Predicting dehulling efficiency of lentils based on seed size and shape characteristics measured with image analysis. Qual. Assur. Saf. Crops Foods. 4, 9–16. doi: 10.1111/j.1757-837X.2011.00119.x

[B55] Shrestha S. Deleuran L. Olesen M. Gislum R. (2015). Use of multispectral imaging in varietal identification of tomato. Sensors 15 (2), 4496–4512. doi: 10.3390/s150204496 25690549 PMC4367422

[B56] Singh A. Dikshit H. K. Mishra G. P. Aski M. Kumar S. (2019). Association mapping for grain diameter and weight in lentil using SSR markers. Plant Gene 20, 100204. doi: 10.1016/j.plgene.2019.100204

[B57] Singh A. Dikshit H. K. Mishra G. P. Aski M. Kumar S. Sarker A. (2022). Breeding for abiotic stress tolerance in lentil in genomic era. In Genomic designing for abiotic stress resistant pulse crops. Ed. Kole C. (Cham: Springer), 145. doi: 10.1007/978-3-030-91039-6_5

[B58] Sonnante G. Hammer K. Pignone D. (2009). From the cradle of agriculture a handful of lentils: history of domestication. Rendiconti. Lincei. 20, 21–37. doi: 10.1007/s12210-009-0002-7

[B59] Stoppel R. Manavski N. Schein A. Schuster G. Teubner M. Schmitz-Linneweber C.. (2012). RHON1 is a novel ribonucleic acid-binding protein that supports RNase e function in the *Arabidopsis* chloroplast. Nucleic Acids Res. 40, 8593–8606. doi: 10.1093/nar/gks613 22735703 PMC3458557

[B60] Teleman A. Tenkanen M. Jacobs A. Dahlman O. (2002). Characterization of O-acetyl-(4-O-methylglucurono) xylan isolated from birch and beech. Carbohydr. Res. 337 (4), 373–377. doi: 10.1016/s0008-6215(01)00327-5 11841818

[B61] Tripathi K. Kumari J. Gore P. G. Mishra D. C. Singh A. K. Mishra G. P.. (2022). Agro-morphological characterization of lentil germplasm of Indian national genebank and development of a core set for efficient utilization in lentil improvement programs. Front. Plant Sci. 12. doi: 10.3389/fpls.2021.751429 PMC8828943 35154171

[B62] Tullu A. Kusmenoglu I. McPhee K. E. Muehlbauer F. J. (2001). Characterization of core collection of lentil germplasm for phenology, morphology, seed and straw yields. Gen. Res. Crop Evol. 48, 143–152. doi: 10.1023/A:1011254629628

[B63] Updegraff D. M. (1969). Semimicro determination of cellulose in biological materials. Anal. Biochem. 32 (3), 420–424. doi: 10.1016/s0003-2697(69)80009-6 5361396

[B64] Verma P. Goyal R. Chahota R. Sharma T. Abdin M. Bhatia S. (2015). Construction of a genetic linkage map and identification of QTLs for seed weight and seed size traits in lentil (L*ens culinaris* medik.). PloS One 10 (10), e0139666. doi: 10.1371/journal.pone.0139666 26436554 PMC4593543

[B65] Verma P. Sharma T. Srivastava P. Abdin M. Bhatia S. (2014). Exploring genetic variability within lentil (*Lens culinaris* medik.) and across related legumes using a newly developed set of microsatellite markers. Mol. Biol. Rep. 41 (9), 5607–5625. doi: 10.1007/s11033-014-3431-z 24893599

[B66] Wang N. (2008). Effect of variety and crude protein content on dehulling quality and on the resulting chemical composition of red lentil (*Lens culinaris*). J. Sci. Food Ag. 88, 885–890. doi: 10.1002/jsfa.3165

[B67] Wang B. Jin S. H. Hu H. Q. Sun Y. G. Wang Y. W. Han P.. (2012). UGT87A2, an *Arabidopsis* glycosyltransferase, regulates flowering time *via* FLOWERING LOCUS c. New Phytol. 194, 666–675. doi: 10.1111/j.1469-8137.2012.04107.x 22404750

[B68] Wang D. Song W. Wei S. Zheng Y. Chen Z. Han J.. (2018). Characterization of the ubiquitin c-terminal hydrolase and ubiquitin-specific protease families in rice (*Oryza sativa*). Front. Plant Sci. 9. doi: 10.3389/fpls.2018.01636 PMC6249995 30498503

[B69] Xu Y. Li H. N. Li G. J. Wang X. Cheng L. G. Zhang Y. M. (2011). Mapping quantitative trait loci for seed size traits in soybean (*Glycine max* l. Merr.). Theor. Appl. Genet. 122, 581–559. doi: 10.1007/s00122-010-1471-x 20981403

[B70] Yang P. Smalle J. Lee S. Yan N. Emborg T. J. Vierstra R. D. (2007). Ubiquitin c-terminal hydrolases 1 and 2 affect shoot architecture in arabidopsis. Plant J. 51 (3), 441–457. doi: 10.1111/j.1365-313X.2007.03154.x 17559514

