# Supplementary figures and images for: Morpho-biochemical characterization of a RIL population for seed parameters and identification of candidate genes regulating seed size trait in lentil (Lens culinaris Medik.)
Source: Front Plant Sci. 2023 Feb 15;14:1091432. doi: 10.3389/fpls.2023.1091432 (PMC9975752; doi:10.3389/fpls.2023.1091432)

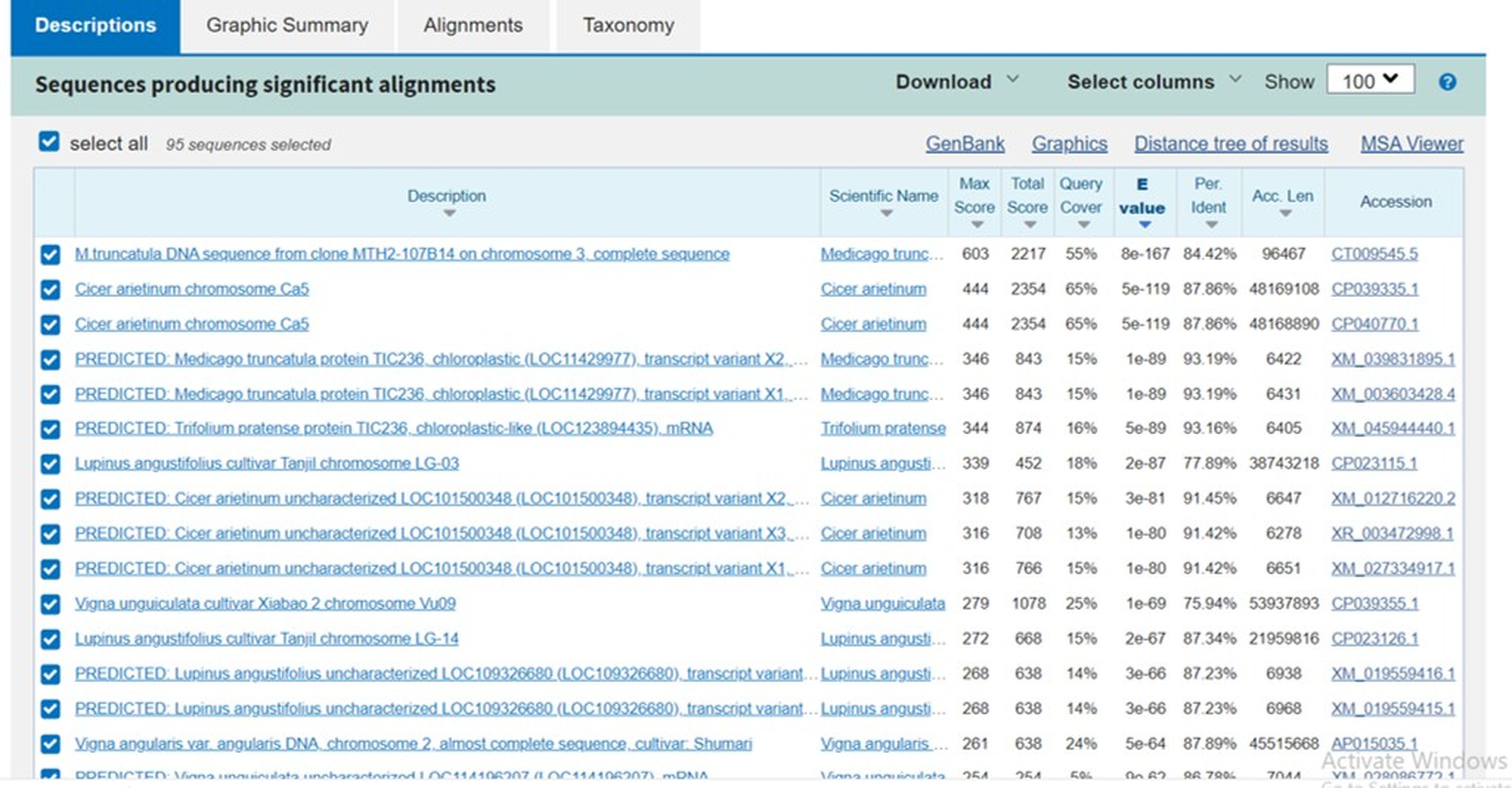

Supplement: Supplementary Figure 1 — Seed size variation among parents (L4602 and L830) and the 10 extreme RILs for large and small seed size. [file Image_1.jpeg]

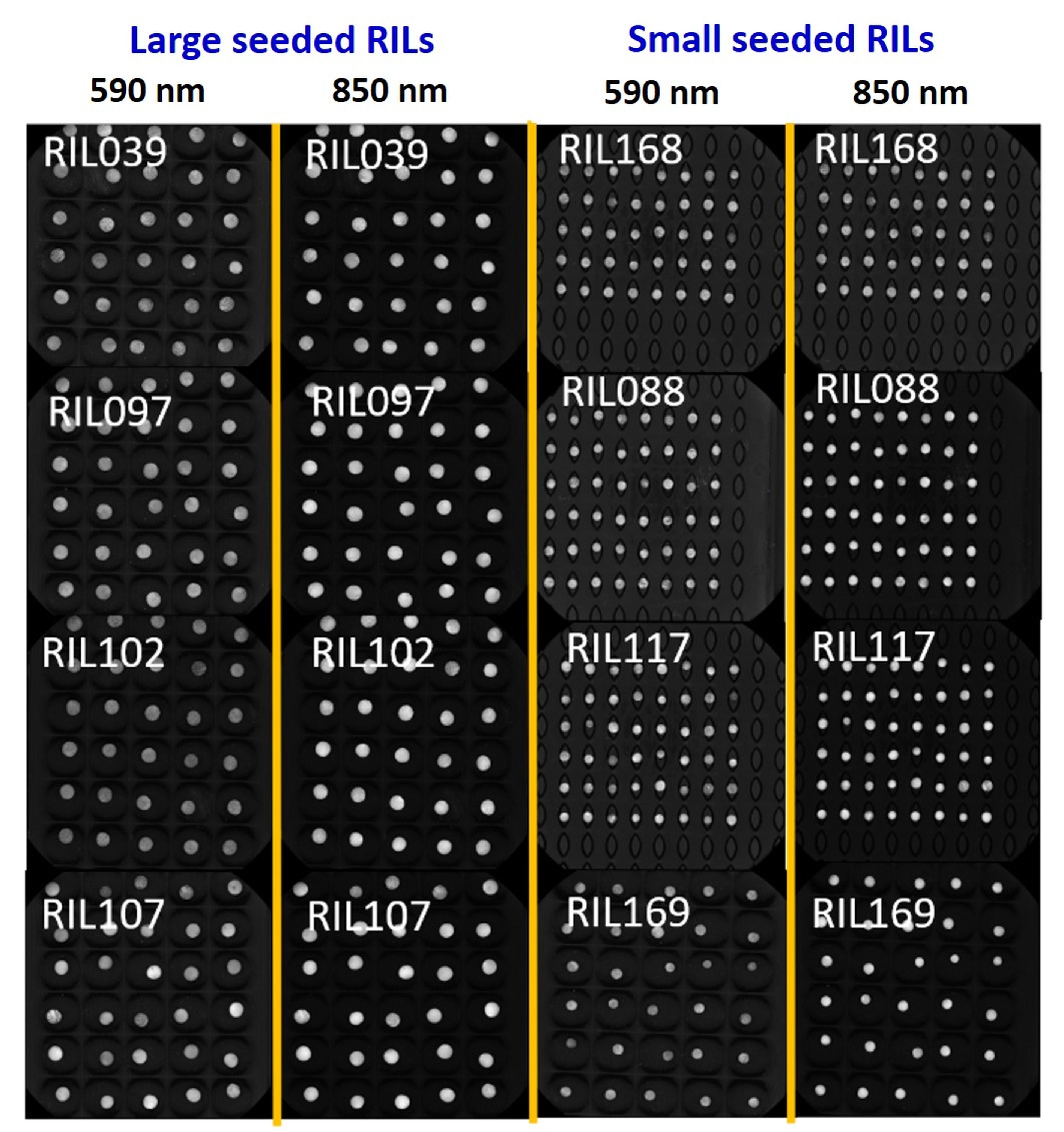

Supplement: Supplementary Figure 2 — A representative gel pictures showing results of parental polymorphism between L4602 and L830 for various SSR markers. Where 1. is L4602 and 2. is L830 [file Image_2.jpeg]

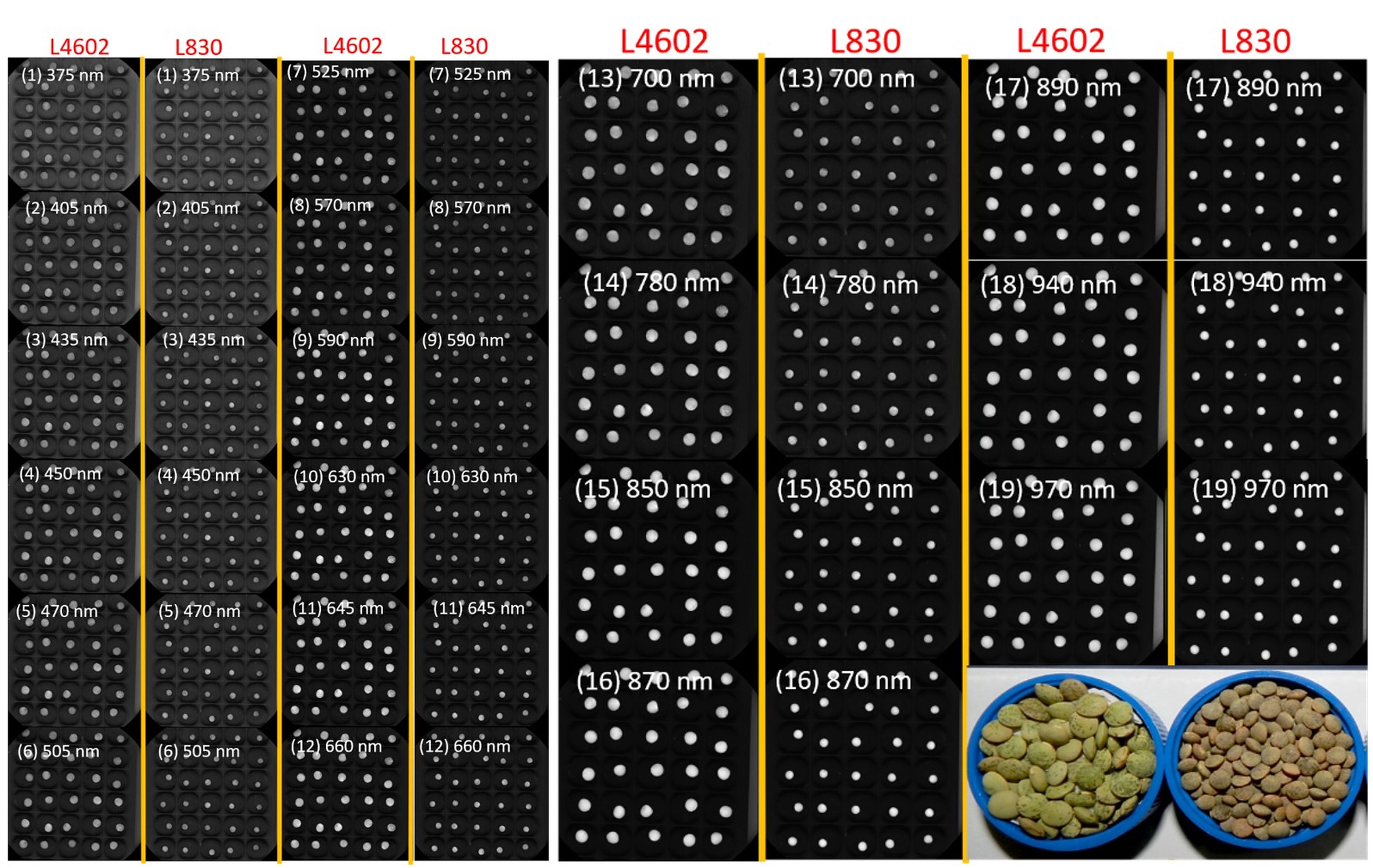

Supplement: Supplementary Figure 3 — BLAST for sequence from large seeded parent (L4602) (149 bp). [file Image_3.jpeg]

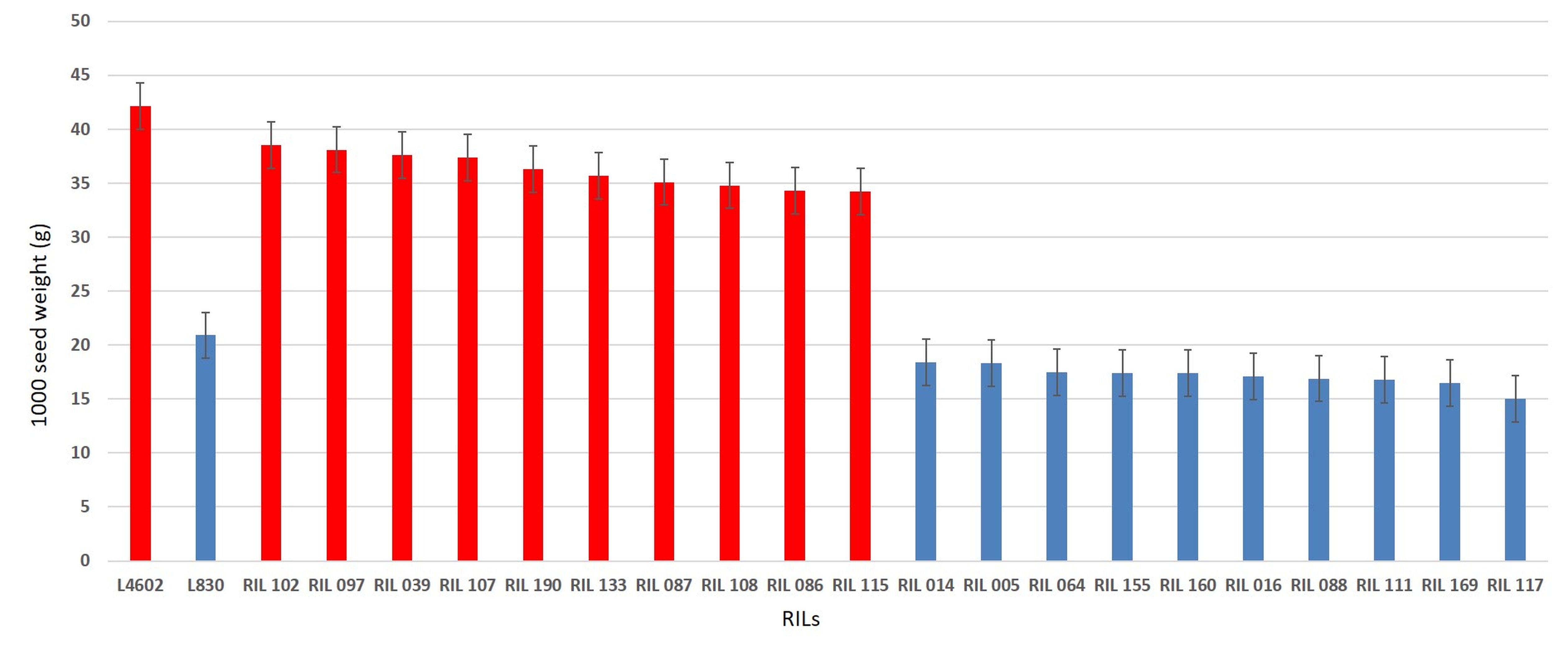

Supplement: Supplementary Figure 4 — BLAST for sequence from small seeded parent (L830) (131 bp; due to 18 bp deletion at two places). [file Image_4.jpeg]

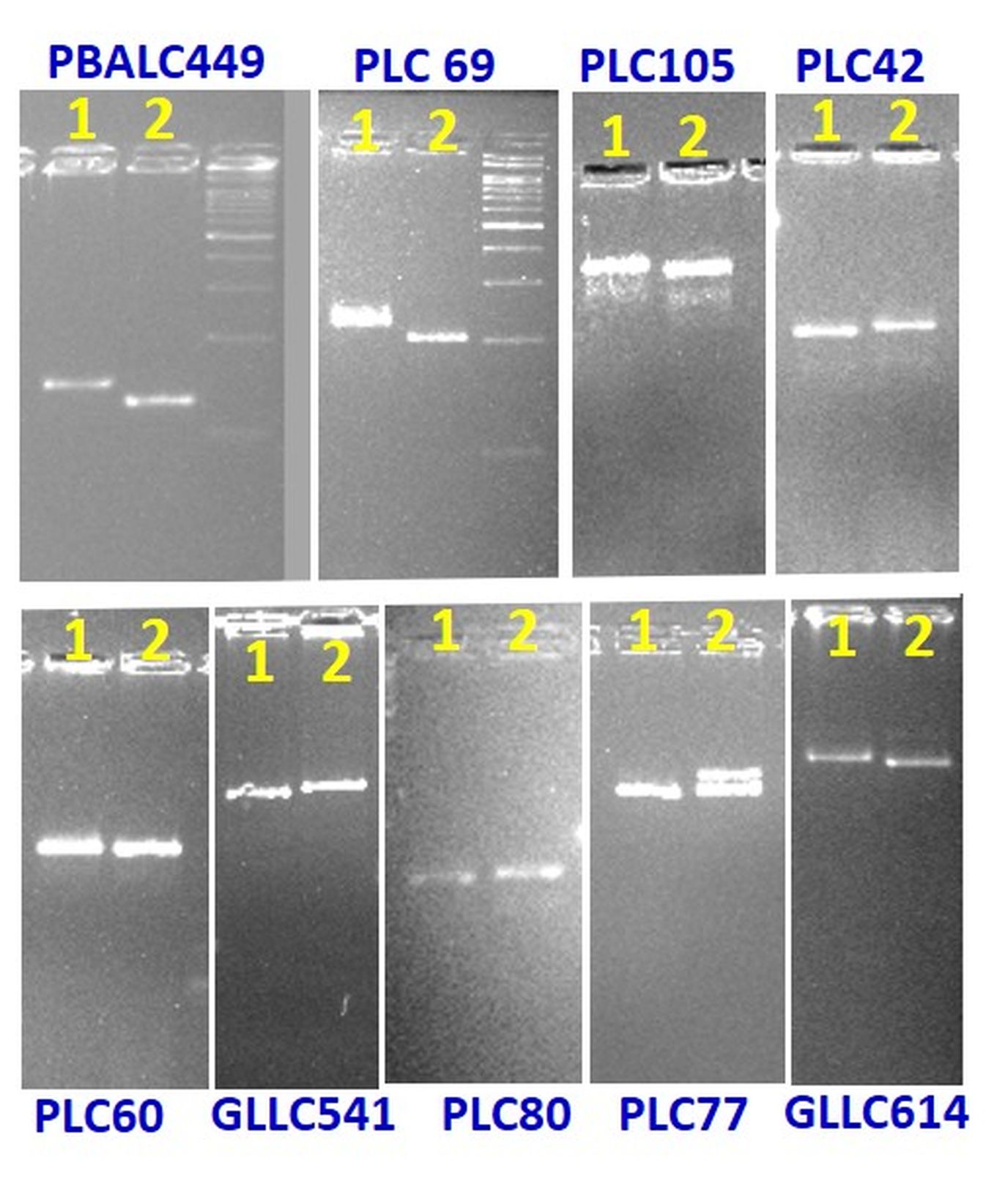

Supplement: Supplementary Figure 5 — Identified position of the SSR (PBALC 449) in the lentil genome (Chromosome 3). [file Image_5.jpeg]

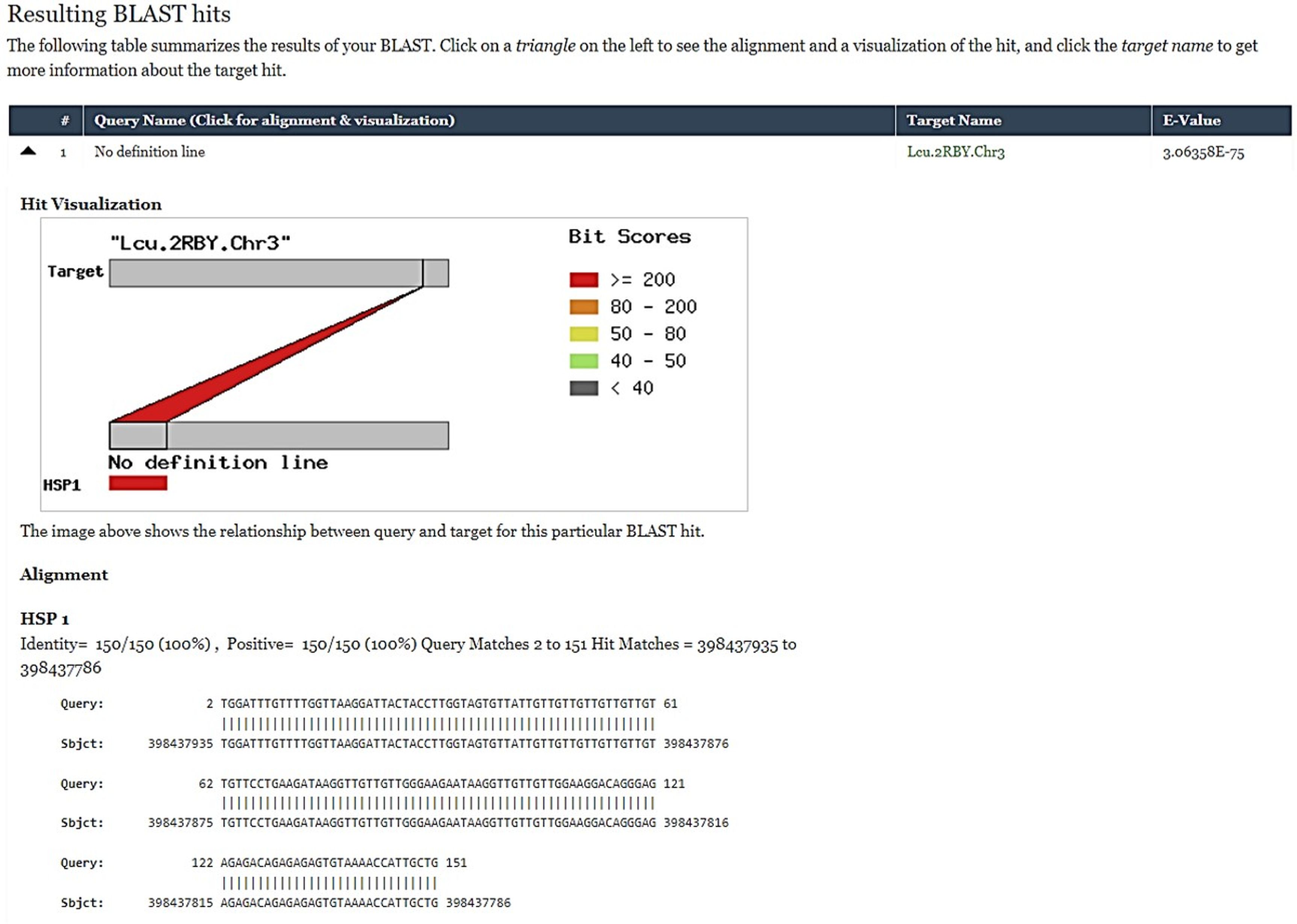

Supplement: Supplementary Figure 6 — Sequnce similarity of the marker (PBALC449) with the relative sp. It is showing most similarity to Medicago truncatula, followed by Cicer arietinum. [file Image_6.jpeg]

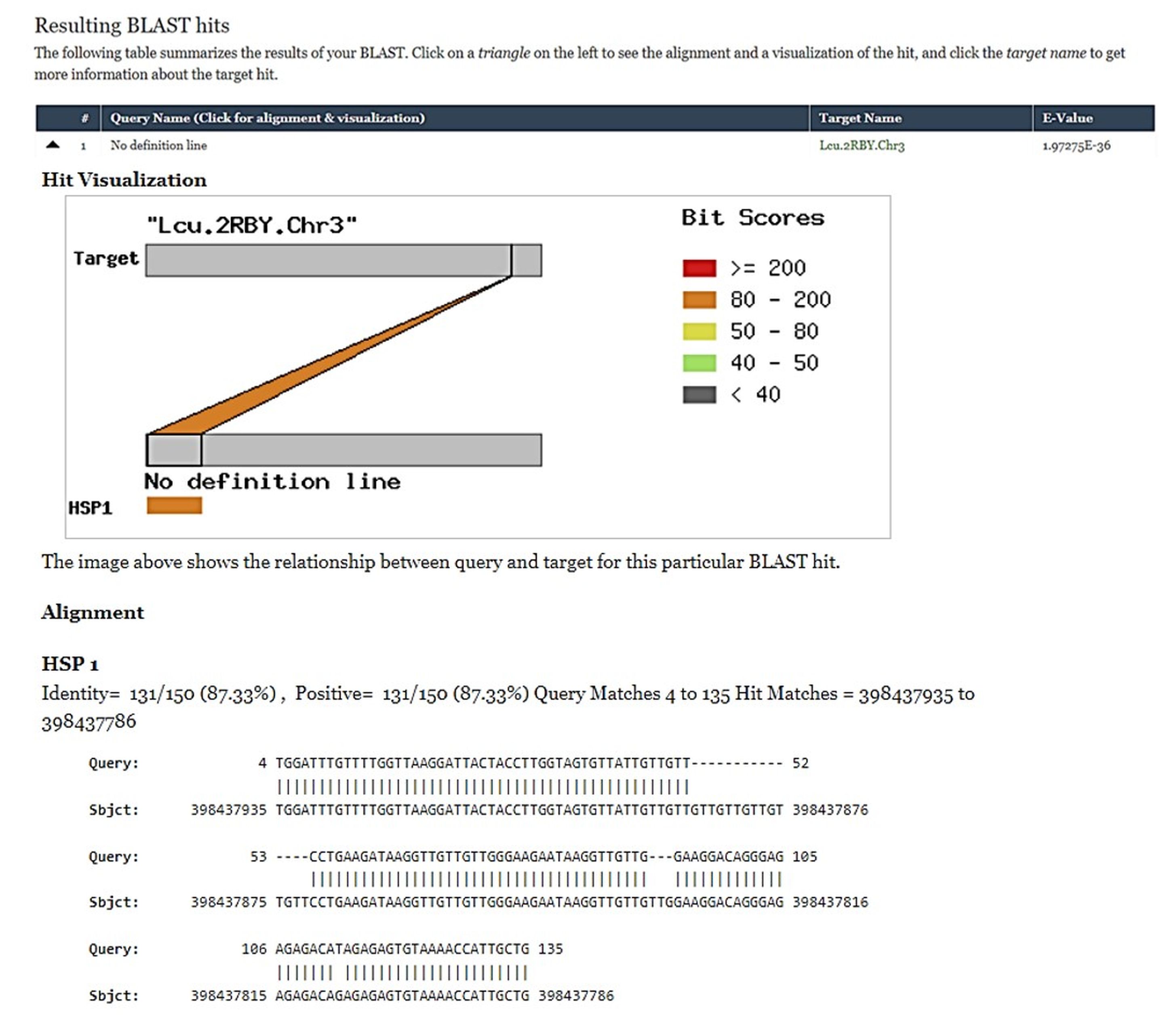

Supplement: Supplementary Figure 7 — Representative image of eight lentil RIL genotypes, captured by VideometerLab 4.0 at two wavelengths (590 and 850 nm) for further seed parameter analysis. [file Image_7.jpeg]

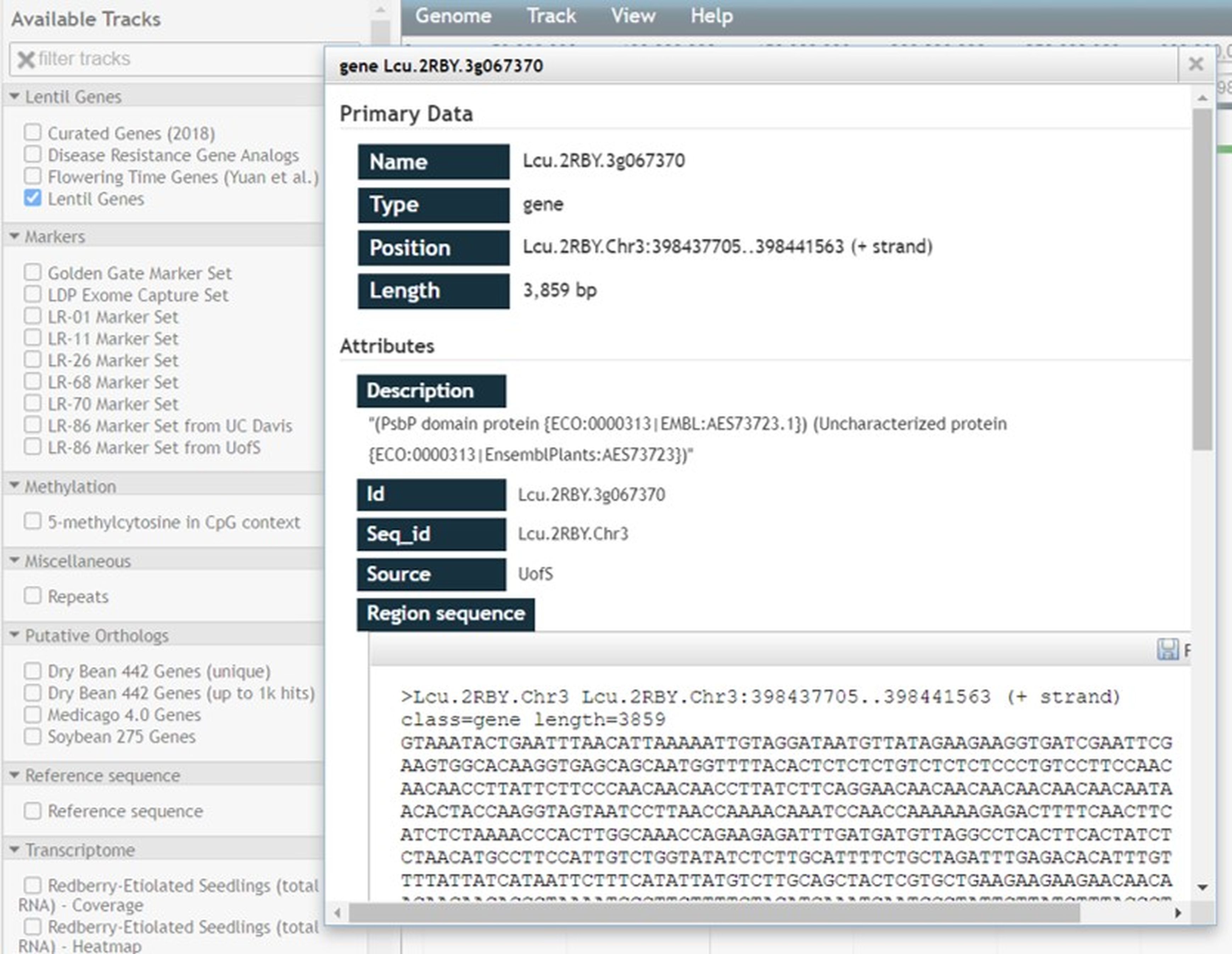

Supplement: Supplementary Figure 8 — Image of the lentil genotypes (L830 and L4602) captured by VideometerLab 4.0 at 19 wavelengths (375 to 970 nm) for further seed parameter analysis. [file Image_8.jpeg]
