# Supplementary material for: Morpho-biochemical characterization of a RIL population for seed parameters and identification of candidate genes regulating seed size trait in lentil (Lens culinaris Medik.)
Source: Front Plant Sci. 2023 Feb 15;14:1091432. doi: 10.3389/fpls.2023.1091432 (PMC9975752; doi:10.3389/fpls.2023.1091432)
Supplement: Supplementary file 9 [file Table_1.docx]

**Table S1. List of primers used for the parental polymorphism survey using L830 and L4602 genotypes.**

| SSRs not/faintly amplified (163 No.) | PBALC (3, 5, 6, 7, 8, 9, 10, 11, 12, 15, 17, 19, 20, 21, 23, 24, 26, 27, 28, 30, 38, 39, 48, 49, 50, 52, 54, 60, 63, 67, 72, 81, 82, 83, 85, 87, 113, 116, 123, 200, PBALC 201, 205, 208, 210, 212, 220, 223, 226, 227, 230, 232, 233, 256, 265, 273, 303, 326, 333, 337, 341, 353, 364, 368, 373, 377, 448, 489, 791, 1409), PLC (1, 2, 3, 5, 6, 7, 8, 9, 14, 19, 23, 24, 25, 47, 49, 56, 57, 61, 63, 67, 68, 71, 73, PLC 75, 84, 86, 89, 90, 91, 92, 94, 97, 99, 100, 102, 106), LC (1, 2, 3, 4, 5, 6, 7, 8, 9, 10, 11, 12, 13, 14, 15, 16, 17, 18, 19, 20, 21, 22, 23, 24, 25, 26, 27, 29, 30, 113, 244, 249, 254, 270, 277, 278, 286, 363, 365, 371, 375, 378, 405, 411, 419, 425, 427), GLLC (106, 511, 527, 556, 559, 587, 591, 592, 595, 598, 609) |
| --- | --- |
| SSRs found monomorphic (200 No.) | PBALC (1, 2, 4, 14, 18, 22, 25, 29, 31, 32, 33, 34, 35, 36, 37, 40, 41, 42, 43, 44, 45, 46, 47, 51, 53, 55, 56, 57, 58, 59, 61, 62, 64, 65, 66, 68, 69, 70, 71, 74, 75, 76, 77, 79, 80, 86, 88, 89, 90, 91, 92, 93, 94, 95, 96, 98, 99, 117, 127, 128, 133, 137, 150, 152, 154, 195, 202, 206, 207, 211, 213, 214, 215, 216, 217, 218, 219, 221, 222, 225, 228, 231, 234, 235, 250, 254, 260, 278, 311, 323, 368, 377, 383, 450, 509, 521, 667, 671, 727, 742, 759, 767), PLC (4, 10, 11, 12, 13, 15, 16, 17, 18, 20, 21, 22, 26, 27, 28, 29, 30, 31, 32, 33, 34, 35, 36, 37, 38, 39, 40, 41, 42, 43, 46, 48, 50, 52, 53, 54, 55, 58, 59, 60, 62, 64, 66, 70, 72, 74, 76, 78, 79, 80, 81, 82, 83, 85, 87, 93, 95, 96, 101, 103, 104), LC (28, 143, 146, 176, 177, 179, 242, 245, 248, 251, 258, 261, 264, 268, 276, 283, 285, 290, 291, 294, 298, 303, 311, 315, 317, 370, 374, 380, 386, 393, 400, 401, 407, 423), GLLC (108, 538, 548) |
| Polymorphic (31 No.) | PBALC (114, 209, 449, 761), PLC (34, 36, 37, 42, 44, 45, 51, 60, 66, 69, 70, 77, 80, 105), LC (272, 301, 305, 307, 385, 389, 396, 398, 421), GLLC (541, 562, 563, 614) |
