# Supplementary material for: Morpho-biochemical characterization of a RIL population for seed parameters and identification of candidate genes regulating seed size trait in lentil (Lens culinaris Medik.)
Source: Front Plant Sci. 2023 Feb 15;14:1091432. doi: 10.3389/fpls.2023.1091432 (PMC9975752; doi:10.3389/fpls.2023.1091432)
Supplement: Supplementary file 10 [file Table_2.docx]

**Table S2. RILs based on seed weight and their pattern of segregation based on PBALC 449 marker.**

| **S. No.** | **RIL No.** | **1000 seed**  **wt (g)** | **Marker**  **(PBALC449)** | **Amplified band**  **Size (bp)** |
| --- | --- | --- | --- | --- |
|  | RIL 117 | 15.0 | P2 | 131bp |
|  | RIL 169 | 16.5 | P2 | 131bp |
|  | RIL 111 | 16.8 | P2 | 131bp |
|  | RIL 088 | 16.9 | P2 | 131bp |
|  | RIL 016 | 17.1 | P2 | 131bp |
|  | RIL 160 | 17.4 | P2 | 131bp |
|  | RIL 155 | 17.4 | P2 | 131bp |
|  | RIL 064 | 17.5 | P2 | 131bp |
|  | RIL 005 | 18.3 | P2 | 131bp |
|  | RIL 014 | 18.4 | P2 | 131bp |
|  | RIL 168 | 18.5 | P2 | 131bp |
|  | RIL 170 | 18.7 | P2 | 131bp |
|  | RIL 140 | 18.7 | P2 | 131bp |
|  | RIL 121 | 18.7 | P2 | 131bp |
|  | RIL 003 | 18.9 | H | 131 & 149bp |
|  | RIL 149 | 18.9 | P2 | 131bp |
|  | RIL 131 | 18.9 | P2 | 131bp |
|  | RIL 002 | 19 | H | 131 & 149bp |
|  | RIL 110 | 19.1 | P2 | 131bp |
|  | RIL 070 | 19.2 | P2 | 131bp |
|  | RIL 010 | 19.2 | P2 | 131bp |
|  | RIL 050 | 19.3 | P2 | 131bp |
|  | RIL 028 | 19.3 | P2 | 131bp |
|  | RIL 063 | 19.4 | H | 131 & 149bp |
|  | RIL 172 | 19.4 | P2 | 131bp |
|  | RIL 161 | 19.4 | P2 | 131bp |
|  | RIL 019 | 19.4 | P2 | 131bp |
|  | RIL 176 | 19.5 | P2 | 131bp |
|  | RIL 118 | 19.5 | P2 | 131bp |
|  | RIL 116 | 19.6 | P2 | 131bp |
|  | RIL 074 | 19.6 | P2 | 131bp |
|  | RIL 067 | 19.6 | P2 | 131bp |
|  | RIL 013 | 19.7 | P1 | 149bp |
|  | RIL 141 | 19.7 | P2 | 131bp |
|  | RIL 076 | 19.7 | P2 | 131bp |
|  | RIL 073 | 19.7 | P2 | 131bp |
|  | RIL 072 | 19.7 | P2 | 131bp |
|  | RIL 175 | 19.9 | P2 | 131bp |
|  | RIL 165 | 19.9 | P2 | 131bp |
|  | RIL 106 | 19.9 | P2 | 131bp |
|  | RIL 183 | 20 | P2 | 131bp |
|  | RIL 123 | 20 | P2 | 131bp |
|  | RIL 105 | 20.1 | H | 131 & 149bp |
|  | RIL 164 | 20.1 | P2 | 131bp |
|  | RIL 137 | 20.1 | P2 | 131bp |
|  | RIL 062 | 20.1 | P2 | 131bp |
|  | RIL 041 | 20.1 | P2 | 131bp |
|  | RIL 009 | 20.1 | P2 | 131bp |
|  | RIL 143 | 20.2 | P2 | 131bp |
|  | RIL 120 | 20.2 | P2 | 131bp |
|  | RIL 109 | 20.2 | P2 | 131bp |
|  | RIL 030 | 20.2 | P2 | 131bp |
|  | RIL 020 | 20.2 | P2 | 131bp |
|  | RIL 139 | 20.3 | P2 | 131bp |
|  | RIL 071 | 20.3 | P2 | 131bp |
|  | RIL 068 | 20.4 | H | 131 & 149bp |
|  | RIL 066 | 20.4 | P1 | 149bp |
|  | RIL 173 | 20.4 | P2 | 131bp |
|  | RIL 052 | 20.4 | P2 | 131bp |
|  | RIL 145 | 20.6 | P2 | 131bp |
|  | RIL 006 | 20.6 | P2 | 131bp |
|  | RIL 124 | 20.7 | P1 | 149bp |
|  | RIL 142 | 20.7 | P2 | 131bp |
|  | RIL 129 | 20.8 | P2 | 131bp |
|  | RIL 021 | 20.9 | P2 | 131bp |
|  | RIL 159 | 21 | P2 | 131bp |
|  | RIL 103 | 21.1 | P2 | 131bp |
|  | RIL 018 | 21.1 | P2 | 131bp |
|  | RIL 080 | 21.2 | H | 131 & 149bp |
|  | RIL 024 | 21.2 | P2 | 131bp |
|  | RIL 079 | 21.3 | H | 131 & 149bp |
|  | RIL 146 | 21.3 | P2 | 131bp |
|  | RIL 075 | 21.4 | P2 | 131bp |
|  | RIL 001 | 21.5 | H | 131 & 149bp |
|  | RIL 034 | 22.1 | P1 | 149bp |
|  | RIL 171 | 22.2 | P2 | 131bp |
|  | RIL 157 | 22.2 | P2 | 131bp |
|  | RIL 130 | 22.3 | P2 | 131bp |
|  | RIL 042 | 22.4 | P1 | 149bp |
|  | RIL 178 | 22.5 | P2 | 131bp |
|  | RIL 069 | 22.6 | H | 131 & 149bp |
|  | RIL 166 | 22.7 | P2 | 131bp |
|  | RIL 156 | 22.8 | P1 | 149bp |
|  | RIL 132 | 22.9 | P2 | 131bp |
|  | RIL 008 | 22.9 | P2 | 131bp |
|  | RIL 163 | 23.2 | P2 | 131bp |
|  | RIL 162 | 23.3 | P2 | 131bp |
|  | RIL 136 | 23.4 | H | 131 & 149bp |
|  | RIL 027 | 23.5 | H | 131 & 149bp |
|  | RIL 158 | 23.5 | P2 | 131bp |
|  | RIL 029 | 23.6 | H | 131 & 149bp |
|  | RIL 138 | 23.8 | H | 131 & 149bp |
|  | RIL 091 | 23.9 | P2 | 131bp |
|  | RIL 147 | 24.0 | H | 131 & 149bp |
|  | RIL 127 | 24.0 | P1 | 149bp |
|  | RIL 049 | 24.0 | P2 | 131bp |
|  | RIL 185 | 24.1 | P1 | 149bp |
|  | RIL 152 | 24.2 | P2 | 131bp |
|  | RIL 181 | 24.3 | P2 | 131bp |
|  | RIL 017 | 24.3 | P2 | 131bp |
|  | RIL 035 | 24.5 | P2 | 131bp |
|  | RIL 025 | 24.6 | P2 | 131bp |
|  | RIL 179 | 24.7 | P2 | 131bp |
|  | RIL 167 | 24.8 | P1 | 149bp |
|  | RIL 012 | 24.8 | P2 | 131bp |
|  | RIL 188 | 24.9 | P1 | 149bp |
|  | RIL 053 | 25.0 | P1 | 149bp |
|  | RIL 186 | 25.2 | P1 | 149bp |
|  | RIL 048 | 25.4 | P2 | 131bp |
|  | RIL 078 | 25.5 | P2 | 131bp |
|  | RIL 154 | 25.6 | P2 | 131bp |
|  | RIL 060 | 25.6 | P2 | 131bp |
|  | RIL 022 | 25.6 | P2 | 131bp |
|  | RIL 144 | 26.0 | H | 131 & 149bp |
|  | RIL 125 | 26.0 | H | 131 & 149bp |
|  | RIL 058 | 26.0 | P2 | 131bp |
|  | RIL 113 | 26.1 | H | 131 & 149bp |
|  | RIL 056 | 26.1 | P1 | 149bp |
|  | RIL 153 | 26.4 | P1 | 149bp |
|  | RIL 061 | 26.6 | P1 | 149bp |
|  | RIL 114 | 27.0 | P1 | 149bp |
|  | RIL 092 | 27.2 | H | 131 & 149bp |
|  | RIL 134 | 27.4 | H | 131 & 149bp |
|  | RIL 096 | 27.5 | P2 | 131bp |
|  | RIL 082 | 27.6 | P1 | 149bp |
|  | RIL 099 | 27.6 | P2 | 131bp |
|  | RIL 101 | 27.7 | P2 | 131bp |
|  | RIL 135 | 27.9 | P1 | 149bp |
|  | RIL 098 | 28.0 | P2 | 131bp |
|  | RIL 182 | 28.1 | H | 131 & 149bp |
|  | RIL 112 | 28.1 | H | 131 & 149bp |
|  | RIL 090 | 28.2 | P1 | 149bp |
|  | RIL 187 | 28.3 | P1 | 149bp |
|  | RIL 023 | 28.7 | H | 131 & 149bp |
|  | RIL 037 | 28.7 | P2 | 131bp |
|  | RIL 057 | 28.8 | P1 | 149bp |
|  | RIL 085 | 28.8 | P2 | 131bp |
|  | RIL 119 | 28.9 | H | 131 & 149bp |
|  | RIL 031 | 29.0 | P2 | 131bp |
|  | RIL 128 | 29.1 | H | 131 & 149bp |
|  | RIL 100 | 29.1 | P1 | 149bp |
|  | RIL 177 | 29.4 | H | 131 & 149bp |
|  | RIL 077 | 29.4 | P1 | 149bp |
|  | RIL 055 | 29.5 | P2 | 131bp |
|  | RIL 036 | 29.5 | P2 | 131bp |
|  | RIL 007 | 29.6 | H | 131 & 149bp |
|  | RIL 150 | 29.8 | P1 | 149bp |
|  | RIL 148 | 29.8 | P1 | 149bp |
|  | RIL 033 | 29.8 | P2 | 131bp |
|  | RIL 004 | 30.3 | H | 131 & 149bp |
|  | RIL 081 | 30.3 | P1 | 149bp |
|  | RIL 174 | 30.4 | H | 131 & 149bp |
|  | RIL 122 | 30.8 | P2 | 131bp |
|  | RIL 040 | 30.9 | P1 | 149bp |
|  | RIL 011 | 30.9 | P1 | 149bp |
|  | RIL 059 | 31.0 | P2 | 131bp |
|  | RIL 189 | 31.3 | H | 131 & 149bp |
|  | RIL 032 | 31.4 | H | 131 & 149bp |
|  | RIL 083 | 31.5 | P1 | 149bp |
|  | RIL 015 | 31.6 | P2 | 131bp |
|  | RIL 038 | 31.7 | H | 131 & 149bp |
|  | RIL 046 | 31.8 | P1 | 149bp |
|  | RIL 180 | 31.8 | P2 | 131bp |
|  | RIL 184 | 32.0 | H | 131 & 149bp |
|  | RIL 089 | 32.0 | H | 131 & 149bp |
|  | RIL 094 | 32.0 | P1 | 149bp |
|  | RIL 095 | 32.2 | P2 | 131bp |
|  | RIL 093 | 32.5 | P1 | 149bp |
|  | RIL 051 | 32.7 | H | 131 & 149bp |
|  | RIL 026 | 32.8 | H | 131 & 149bp |
|  | RIL 084 | 33.1 | H | 131 & 149bp |
|  | RIL 104 | 33.2 | P1 | 149bp |
|  | RIL 044 | 33.2 | P1 | 149bp |
|  | RIL 045 | 33.3 | H | 131 & 149bp |
|  | RIL 065 | 33.5 | H | 131 & 149bp |
|  | RIL 054 | 33.6 | H | 131 & 149bp |
|  | RIL 043 | 33.8 | H | 131 & 149bp |
|  | RIL 047 | 34.0 | H | 131 & 149bp |
|  | RIL 115 | 34.2 | P1 | 149bp |
|  | RIL 086 | 34.3 | P1 | 149bp |
|  | RIL 108 | 34.8 | P2 | 131bp |
|  | RIL 087 | 35.1 | P1 | 149bp |
|  | RIL 133 | 35.7 | P1 | 149bp |
|  | RIL 190 | 36.3 | H | 131 & 149bp |
|  | RIL 107 | 37.4 | P2 | 131bp |
|  | RIL 039 | 37.6 | H | 131 & 149bp |
|  | RIL 097 | 38.1 | P1 | 149bp |
|  | RIL 102 | 38.5 | P2 | 131bp |
|  | RIL 126 | Lost during development | Lost during development | - |
|  | RIL 151 | Lost during development | Lost during development | - |
|  | L4602 | 43.4 | P1 | 149bp |
|  | L830 | 19.7 | P2 | 131bp |
