# Supplementary material for: Morpho-biochemical characterization of a RIL population for seed parameters and identification of candidate genes regulating seed size trait in lentil (Lens culinaris Medik.)
Source: Front Plant Sci. 2023 Feb 15;14:1091432. doi: 10.3389/fpls.2023.1091432 (PMC9975752; doi:10.3389/fpls.2023.1091432)
Supplement: Supplementary file 11 [file Table_3.docx]

**Table S3. Sequence details of the P1 (L4602) and P2 (L830) band as amplified by the SSR marker PBALC 449.**

| **Sequence of the SSR (PBALC 449) product for the large seeded parent (L4602) (149 bp)** | **Sequence of the SSR (PBALC 449) product for the small seeded parent (L830) (131 bp)** |
| --- | --- |
| ATGGATTTGTTTTGGTTAAGGATTACTACCTTGGTAGTGTTATTGTTGTTGTTGTTGTTGTTGTTCCTGAAGATAAGGTTGTTGTTGGGAAGAATAAGGTTGTTGTTGGAAGGACAGGGAGAGAGACAGAGAGAGTGTAAAACCATTGCTGATCTTTCTAGAAGATCTCCTACAATATTCTCAGCTGCCATGGAAAATCGATGTTCTTCTTTTATTCTCTCAAGATTTTCAGGCTGTATATTAAAACTTATATTAAGAACTATGCTAACCACCTCATCAGGAACCGTTGTAGGTGGCGTGGGTTTTCTTGGCAATCGACTCTCATGAAAACTACGAGCTAAATATTCAATATGTTCCTCTTGACCAACTTTATTCTGCATTTTTTTTGAACGAGGTTTAGAGCAAGCTTCAGGAAACTGAGACAGGAATTTTATTAAAAATTTAAATTTTGAAGAAAGTTCAGGGTTAATAGCATCCATTTTTTGCTTTGCAAGTTCCTCAGCATTCTTAACAAAAGACGTCTCTTTTGACATGTTTAAAGTTTAAACCTCCTGTGTGAAATTATTATCCGCTCATAATTCCACACATTATACGAGCCGGAAGCATAAAGTGTAAAGCCTGGGGTGCCTAATGAGTGAGCTAACTCACATTAATTGCGTTGCGCTCACTGCCAATTGCTTTCCAGTCGGGAAACCTGTCGTGCCAGCTGCATTAATGAATCGGCCAACGCGCGGGGAGAGGCGGTTTGCGTATTGGGCGCTCTTCCGCTTCCTCGCTCACTGACTCGCTGCGCTCGGTCGTTCGGCTGCGGCGAGCGGTATCAGCTCACTCAAAGGCGGTAATACGGTTATCCACAGAATCAGGGATAACGCAGGAAAGAACATGTGA | AGATGGATTTGTTTTGGTTAAGGATTACTACCTTGGTAGTGTTATTGTTGTTCCTGAAGATAAGGTTGTTGTTGGGAAGAATAAGGTTGTTGGAAGGACAGGGAGAGAGACATAGAGAGTGTAAAACCATTGCTGATCTTTCTAGAAGATCTCCTACAATATTCTCAGCTGCCATGGAAAATCGATGTTCTTCTTTTATTCTCTCAAGATTTTCAGGCTGTATATTAAAACTTATATTAAGAACTATGCTAACCACCTCATCAGGAACCGTTGTAGGTGGCGTGGGTTTTCTTGGCAATCGACTCTCATGAAAACTACGAGCTAAATATTCAATATGTTCCTCTTGACCAACTTTATTCTGCATTTTTTTTGAACGAGGTTTAGAGCAAGCTTCAGGAAACTGAGACAGGAATTTTATTAAAAATTTAAATTTTGAAGAAAGTTCAGGGTTAATAGCATCCATTTTTTGCTTTGCAAGTTCCTCAGCATTCTTAACAAAAGACGTCTCTTTTGACATGTTTAAAGTTTAAACCTCCTGTGTGAAATTATTATCCGCTCATAATTCCACACATTATACGAGCCGGAAGCATAAAGTGTAAAGCCTGGGGTGCCTAATGAGTGAGCTAACTCACATTAATTGCGTTGCGCTCACTGCCAATTGCTTTCCAGTCGGGAAACCTGTCGTGCCAGCTGCATTAATGAATCGGCCAACGCGCGGGGAGAGGCGGTTTGCGTATTGGGCGCTCTTCCGCTTCCTCGCTCACTGACTCGCTGCGCTCGGTCGTTCGGCTGCGGCGAGCGGTATCAGCTCACTCAAAGGCGGTAATACGGTTATCCACAGAATCAGGGGATAACGCATGAAAGAACATGTGAGCAAAAGGCCAGCAAAAGGCCAGGAACCGTAAAAAGGCCGCGTTGCTGGCGTTTTTCCATAGGCTCCGCCCCCCTGA |
