# Supplementary material for: Morpho-biochemical characterization of a RIL population for seed parameters and identification of candidate genes regulating seed size trait in lentil (Lens culinaris Medik.)
Source: Front Plant Sci. 2023 Feb 15;14:1091432. doi: 10.3389/fpls.2023.1091432 (PMC9975752; doi:10.3389/fpls.2023.1091432)
Supplement: Supplementary file 12 [file Table_4.docx]

**Table S4. Grouping of 188 RILs and parents for different seed parameter (one way ANOVA).**

| **P1 (L4602)** | **<P1 and >P2** | **P2 (L830)** | < **P2** |
| --- | --- | --- | --- |
| **Area (mm2)** |  |  |  |
| **A** | **B to Z; AA to AZ; BA to BD** | **BE to BT** | **BU to BY** |
| L4602 | L004, L007, L011, L012, L015, L017, L022, L023, L025, L026, L027, L028, L029, L031, L032, L033, L035, L036, L037, L038, L039, L040, L042, L043, L044, L045, L046, L047, L048, L049, L051, L054, L055, L056, L057, L058, L059, L060, L061, L065, L077, L078, L081, L082, L083, L084, L085, L086, L087, L089, L090, L092, L093, L094, L095, L096, L097, L098, L099, L100, L101, L102, L104, L107, L108, L112, L113, L114, L115, L119, L122, L125, L127, L128, L132, L133, L134, L135, L136, L138, L144, L147, L148, L150, L152, L153, L154, L158, L162, L167, L171, L174, L177, L179, L180, L181, L182, L183, L184, L185, L187, L188, L189, L190 | L001, L003, L005, L006, L008, L010, L013, L014, L016, L018, L019, L020, L021, L024, L030, L034, L041, L050, L052, L053, L062, L063, L066, L067, L068, L069, L070, L071, L072, L073, L074, L075, L076, L079, L080, L091, L103, L105, L106, L109, L110, L116, L118, L120, L121, L124, L129, L130, L131, L137, L139, L140, L141, L142, L143, L145, L146, L149, L155, L156, L157, L159, L160, L161, L163, L164, L165, L166, L168, L169, L170, L172, L173, L175, L176, L178, **L830** | L002, L064, L088, L111, L117 |
| **Seed wt (g)** |  |  |  |
| **A** | **B to Z; AA to AW** | **AX to AZ; BB to BS** | **BT BU** |
| L4602, L039, L102, L107 | L004, L007, L011, L012, L015, L023, L026, L027, L031, L032, L033, L036, L037, L038, L040, L043, L044, L045, L046, L047, L048, L049, L051, L053, L054, L055, L056, L057, L058, L059, L061, L065, L077, L078, L081, L082, L083, L084, L085, L086, L087, L089, L090, L092, L093, L094, L095, L096, L097, L098, L099, L100, L101, L104, L108, L112, L113, L114, L115, L119, L122, L125, L127, L128, L133, L134, L135, L136, L144, L147, L148, L150, L153, L167, L174, L177, L179, L180, L182, L184, L186, L187, L189, L190 | L001, L002, L003, L005, L006, L008, L009, L010, L013, L014, L016, L017, L018, L019, L020, L021, L022, L024, L025, L028, L029, L030, L034, L035, L041, L042, L050, L052, L060, L062, L063, L064, L066, L067, L068, L069, L070, L071, L072, L073, L074, L075, L076, L079, L080, L088, L091, L103, L105, L106, L109, L110, L111, L116, L118, L120, L121, L123, L124, L129, L130, L131, L132, L137, L138, L139, L140, L141, L142, L143, L145, L146, L149, L152, L154, L155, L156, L157, L158, L159, L160, L161, L162, L163, L164, L165, L166, L168, L170, L171, L172, L173, L175, L176, L178, L181, L183, L185, L188, L830 | L117, L169 |
| **Length (mm)** |  |  |  |
| **A** | **B to Z; AA to AZ; BB to BN** | **BO to BZ** | **CA to CD** |
| L4602 | L004, L007, L008, L011, L012, L015, L017, L022, L023, L025, L026, L027, L028, L029, L031, L032, L033, L035, L036, L037, L038, L039, L040, L042, L043, L044, L045, L046, L047, L048, L049, L051, L053, L054, L055, L056, L057, L058, L059, L060, L061, L065, L077, L078, L079, L081, L082, L083, L084, L085, L086, L087, L089, L090, L092, L093, L094, L095, L096, L097, L098, L099, L100, L101, L102, L104, L107, L108, L112, L113, L114, L115, L119, L122, L125, L127, L128, L132, L133, L134, L135, L136, L138, L144, L146, L147, L148, L150, L152, L153, L154, L156, L157, L158, L162, L163, L166, L167, L171, L174, L175, L177, L179, L180, L181, L182, L183, L184, L185, L187, L188, L189, L190 | L001, L003, L005, L006, L010, L013, L014, L016, L018, L019, L020, L021, L024, L030, L034, L041, L050, L052, L062, L063, L064, L066, L067, L068, L069, L070, L071, L072, L073, L074, L075, L076, L080, L091, L103, L105, L106, L109, L110, L116, L118, L120, L121, L124, L129, L130, L131, L137, L139, L140, L141, L142, L143, L145, L149, L155, L159, L160, L161, L164, L165, L168, L169, L170, L172, L173, L176, L178, L830 | L002, L088, L111, L117 |
| **Width (mm)** |  |  |  |
| **A** | **B to Z; AA to AZ; BB to BD** | **BE to BR** | **BS to BY** |
| L097, L108, L133, L4602 | L004, L007, L011, L012, L015, L017, L022, L023, L025, L026, L027, L029, L031, L032, L033, L035, L036, L037, L038, L039, L040, L042, L043, L044, L045, L046, L047, L048, L049, L051, L054, L055, L056, L057, L058, L059, L060, L061, L065, L077, L078, L081, L082, L083, L084, L085, L086, L087, L089, L090, L092, L093, L094, L095, L096, L098, L099, L100, L101, L102, L104, L107, L112, L113, L114, L115, L119, L122, L125, L127, L128, L134, L135, L136, L138, L144, L147, L148, L150, L152, L153, L154, L158, L162, L167, L171, L174, L177, L179, L180, L181, L182, L183, L184, L185, L187, L188, L189, L190 | L001, L003, L005, L006, L008, L010, L013, L014, L016, L018, L019, L020, L021, L024, L028, L030, L034, L041, L050, L052, L053, L062, L063, L066, L067, L068, L069, L071, L072, L074, L075, L076, L079, L080, L091, L103, L105, L106, L109, L110, L116, L118, L120, L121, L124, L129, L130, L131, L132, L137, L139, L141, L142, L143, L145, L146, L149, L155, L156, L157, L159, L160, L161, L163, L164, L166, L168, L169, L170, L172, L173, L175, L176, L178, L830 | L002, L064, L070, L073, L088, L111, L117, L140, L165 |
| **Ratio Width Length** |  |  |  |
|  |  | **A to D** | **E to R** |
|  |  | L001, L002, L003, L004, L005, L006, L007, L010, L011, L012, L013, L014, L015, L016, L017, L018, L019, L020, L021, L022, L023, L024, L025, L026, L027, L028, L029, L030, L032, L033, L034, L035, L036, L037, L038, L039, L040, L041, L044, L045, L046, L047, L048, L049, L050, L052, L053, L054, L055, L056, L057, L058, L059, L060, L061, L062, L063, L064, L065, L067, L068, L069, L070, L071, L072, L073, L074, L075, L076, L077, L078, L079, L080, L081, L082, L084, L085, L087, L089, L090, L092, L095, L096, L097, L098, L099, L100, L101, L102, L103, L105, L106, L107, L108, L109, L110, L111, L112, L113, L115, L116, L117, L118, L119, L120, L121, L122, L124, L125, L127, L128, L129, L130, L131, L133, L134, L135, L136, L137, L138, L139, L140, L141, L142, L143, L144, L145, L146, L147, L148, L149, L150, L152, L153, L154, L155, L156, L157, L158, L160, L161, L162, L164, L165, L166, L167, L168, L169, L170, L171, L172, L173, L176, L177, L178, L179, L182, L183, L184, L185, L187, L189, L190, L4602, L830 | L008, L031, L042, L043, L051, L066, L083, L086, L088, L091, L093, L094, L104, L114, L132, L159, L163, L174, L175, L180, L181, L188 |
| **Compactness Circle** |  |  |  |
|  |  | **A to G** | **H to V** |
|  |  | L001, L002, L003, L004, L005, L006, L007, L010, L011, L012, L013, L014, L015, L016, L017, L018, L019, L020, L021, L022, L023, L024, L025, L026, L027, L028, L029, L030, L032, L033, L034, L035, L036, L037, L038, L039, L040, L041, L043, L044, L045, L046, L047, L048, L049, L050, L052, L053, L054, L055, L056, L057, L058, L059, L060, L061, L062, L063, L064, L065, L067, L068, L069, L070, L071, L072, L073, L074, L075, L076, L077, L078, L079, L080, L081, L082, L084, L085, L087, L089, L090, L092, L095, L096, L097, L098, L099, L100, L101, L102, L103, L105, L106, L107, L108, L109, L110, L111, L112, L113, L114, L115, L116, L117, L118, L119, L120, L121, L122, L124, L125, L127, L128, L129, L130, L131, L133, L134, L135, L136, L137, L138, L139, L140, L141, L142, L143, L144, L145, L146, L147, L148, L149, L150, L152, L153, L154, L155, L156, L157, L158, L160, L161, L162, L164, L165, L166, L167, L168, L169, L170, L171, L172, L173, L176, L177, L178, L179, L182, L183, L184, L187, L189, L190, L4602, L830 | L008, L031, L042, L051, L066, L083, L086, L088, L091, L093, L094, L104, L132, L159, L163, L174, L175, L180, L181, L185, L188 |
| **Ratio (Width/ Area)** |  |  | **>L830** |
| **BQ to BS** | **T to Z; AA to AZ; BB to BP** | **B to S** | **A** |
| L039, L097, L133, L4602 | L004, L007, L008, L011, L012, L015, L017, L022, L023, L025, L026, L027, L028, L029, L031, L032, L033, L035, L036, L037, L038, L040, L042, L043, L044, L045, L046, L047, L048, L049, L051, L053, L054, L055, L056, L057, L058, L059, L060, L061, L065, L077, L078, L079, L080, L081, L082, L083, L084, L085, L086, L087, L089, L090, L092, L093, L094, L095, L096, L098, L099, L100, L101, L102, L104, L107, L108, L112, L113, L114, L115, L119, L122, L125, L127, L128, L132, L134, L135, L136, L138, L144, L146, L147, L148, L150, L152, L153, L154, L156, L157, L158, L162, L163, L166, L167, L171, L174, L177, L179, L180, L181, L182, L183, L184, L185, L187, L188, L189, L190 | L001, L003, L005, L006, L010, L013, L014, L016, L018, L019, L020, L021, L024, L030, L034, L041, L050, L052, L062, L063, L066, L067, L068, L069, L070, L071, L072, L073, L074, L075, L076, L091, L105, L106, L109, L110, L116, L118, L120, L121, L124, L129, L130, L131, L137, L139, L140, L141, L142, L143, L145, L149, L155, L159, L160, L161, L164, L165, L168, L169, L170, L172, L173, L175, L176, L178, L830 | L002, L064, L088, L103, L111, L11 |
| **Volume** |  |  |  |
| **A** | **B to Z; AA to AZ; BB to BN** | **BO to BZ** | **CA to CD** |
| L4602 | L004, L007, L008, L011, L012, L015, L017, L022, L023, L025, L026, L027, L028, L029, L031, L032, L033, L035, L036, L037, L038, L039, L040, L042, L043, L044, L045, L046, L047, L048, L049, L051, L053, L054, L055, L056, L057, L058, L059, L060, L061, L065, L077, L078, L079, L081, L082, L083, L084, L085, L086, L087, L089, L090, L092, L093, L094, L095, L096, L097, L098, L099, L100, L101, L102, L104, L107, L108, L112, L113, L114, L115, L119, L122, L125, L127, L128, L132, L133, L134, L135, L136, L138, L144, L146, L147, L148, L150, L152, L153, L154, L156, L157, L158, L162, L163, L166, L167, L171, L174, L175, L177, L179, L180, L181, L182, L183, L184, L185, L187, L188, L189, L190 | L001, L003, L005, L006, L010, L013, L014, L016, L018, L019, L020, L021, L024, L030, L034, L041, L050, L052, L062, L063, L064, L066, L067, L068, L069, L070, L071, L072, L073, L074, L075, L076, L080, L091, L103, L105, L106, L109, L110, L116, L118, L120, L121, L124, L129, L130, L131, L137, L139, L140, L141, L142, L143, L145, L149, L155, L159, L160, L161, L164, L165, L168, L169, L170, L172, L173, L176, L178, L830 | L002, L088, L111, L117 |
| **Perimeter** |  |  |  |
| **A** | **B to Z; AA to AZ; BB to BC** | **BD to BS** | **BT to BX** |
| L4602 | L004, L007, L008, L011, L012, L015, L017, L022, L023, L025, L026, L027, L028, L029, L031, L032, L033, L035, L036, L037, L038, L039, L040, L042, L043, L044, L045, L046, L047, L048, L049, L051, L053, L054, L055, L056, L057, L058, L059, L060, L061, L065, L077, L078, L081, L082, L083, L084, L085, L086, L087, L089, L090, L092, L093, L094, L095, L096, L097, L098, L099, L100, L101, L102, L104, L107, L108, L112, L113, L114, L115, L119, L122, L125, L127, L128, L132, L133, L134, L135, L136, L138, L144, L147, L148, L150, L152, L153, L154, L158, L162, L167, L171, L174, L177, L179, L180, L181, L182, L183, L184, L185, L187, L188, L189, L190 | L001, L003, L005, L006, L010, L013, L014, L016, L018, L019, L020, L021, L024, L030, L034, L041, L050, L052, L062, L063, L066, L067, L068, L069, L070, L071, L072, L073, L074, L075, L076, L079, L080, L091, L103, L105, L106, L109, L110, L116, L118, L120, L121, L124, L129, L130, L131, L137, L139, L140, L141, L142, L143, L145, L146, L149, L155, L156, L157, L159, L160, L161, L163, L164, L165, L166, L168, L169, L170, L172, L173, L175, L176, L178, L830 | L002, L064, L088, L111, L117 |
