# Supplementary material for: Morpho-biochemical characterization of a RIL population for seed parameters and identification of candidate genes regulating seed size trait in lentil (Lens culinaris Medik.)
Source: Front Plant Sci. 2023 Feb 15;14:1091432. doi: 10.3389/fpls.2023.1091432 (PMC9975752; doi:10.3389/fpls.2023.1091432)
Supplement: Supplementary file 13 [file Table_5.docx]

**Table S5. Identification of SNPs/InDels in putative candidate genes in the genomic region for seed siz on Lcu.2RBY.Chr3 (Chromosome number 3)**

| **Gene (Gene ID)** | **Position (bp)** | **Parent (Globe mutant) Late** | **Late bulk (base)** | **Early bulk (base)** | **ΔSNP index** | **Annotation on base** | **Effect type** | **Effects by impact** |
| --- | --- | --- | --- | --- | --- | --- | --- | --- |
| ***E3 ubiqutin ligase (Lcu.2RBY.3g067340)*** | 398420753 | G | G | A | 1 | ANN=A | intron_variant | **MODIFIER** |
|  | 398422292 | A | A | C | 1 | ANN=C | upstream_gene_variant |  |
|  | 398423374 | T | T | G | 1 | ANN=T | 3_prime_UTR_variant |  |
| ***TIFY-like protein***  ***(Lcu.2RBY.3g067310)*** | 398346714 | G | G | A | 1 | ANN=G | **3_prime_UTR_variant** | **MODIFIER** |
|  | 398346857 | T | T | C | 1 | ANN=T |  |  |
|  | 398346875 | A | A | G | 1 | ANN=A |  |  |
|  | 398346906 | C | C | T | 1 | ANN=C |  |  |
|  | 398346966 | A | A | T | 1 | ANN=A |  |  |
|  | 398347009 | T | T | A | 1 | ANN=T |  |  |
|  | 398347038 | G | G | A | 1 | ANN=G |  |  |
|  | 398347151 | C | C | T | 1 | ANN=C |  |  |
|  | 398347291 | T | T | C | 1 | ANN=T |  |  |
|  | 398347688 | A | A | G | 1 | ANN=A | **upstream_gene_variant** |  |
|  | 398347691 | G | G | A | 1 | ANN=G |  |  |
|  | 398347790 | G | G | C | 1 | ANN=G |  |  |
|  | 398347875 | C | C | G | 1 | ANN=C |  |  |
|  | 398347885 | G | G | A | 1 | ANN=G |  |  |
|  | 398347897 | A | A | G | 1 | ANN=A |  |  |
|  | 398347916 | G | G | A | 1 | ANN=G |  |  |
|  | 398347985 | A | A | G | 1 | ANN=G |  |  |
|  | 398348454 | T | T | C | 1 | ANN=T |  |  |
|  | 398348534 | T | T | A | 1 | ANN=T |  |  |
|  | 398348539 | C | C | T | 1 | ANN=C |  |  |
|  | 398349040 | T | T | C | 1 | ANN=T | **intron_variant** |  |
|  | 398349118 | AGGGGG | AGGGGGGG | AGGGGG | 1 | ANN=AGGGGGGG |  |  |
|  | 398349204 | C | C | T | 1 | ANN=C |  |  |
|  | 398349222 | A | A | G | 1 | ANN=A |  |  |
|  | 398349276 | C | C | T | 1 | ANN=C |  |  |
|  | 398349353 | A | A | G | 1 | ANN=A |  |  |
|  | 398349648 | C | C | A | 1 | ANN=C |  |  |
|  | 398349676 | T | T | C | 1 | ANN=T |  |  |
|  | 398349691 | T | T | C | 1 | ANN=T |  |  |
|  | 398349704 | A | A | T | 1 | ANN=A |  |  |
|  | 398349708 | T | T | C | 1 | ANN=T |  |  |
|  | 398349715 | T | T | G | 1 | ANN=T |  |  |
|  | 398351336 | A | A | G | 1 | ANN=A | synonymous_variant | **LOW** |
|  | 398351395 | G | G | A | 1 | ANN=G | **5_prime_UTR_variant** | **MODIFIER** |
|  | 398351396 | C | C | T | 1 | ANN=T |  |  |
| ***Hexosyltransferase (Lcu.2RBY.3g067290)*** | 398337805 | A | A | T | 1 | ANN=A |  | **MODIFIER** |
|  | 398337867 | T | T | G | 1 | ANN=T |  |  |
|  | 398337868 | G | G | A | 1 | ANN=G |  |  |
|  | 398337877 | G | G | A | 1 | ANN=G |  |  |
|  | 398337906 | G | G | A | 1 | ANN=G | 5_prime_UTR_premature_start_codon_gain_variant | **LOW** |
|  | 398337992 | ATTCTTCTTCTTCTTCTTCT | ATTCTTCTTCTTCTTCT | ATTCTTCTTCTTCTTCTTCT | 1 | ANN=ATTCTTCTTCTTCTTCT | disruptive_inframe_deletion | **MODERATE** |
|  | 398338030 | T | T | A | 1 | ANN=T | **downstream_gene_variant** | **MODIFIER** |
|  | 398338083 | C | C | T | 1 | ANN=T |  |  |
|  | 398338101 | C | C | G | 1 | ANN=C |  |  |
|  | 398338164 | C | C | T | 1 | ANN=C |  |  |
|  | 398338179 | A | A | T | 1 | ANN=T |  |  |
|  | 398338272 | TCTTACTTA | TCTTACTTA | TCTTA | 1 | ANN=TCTTA |  |  |
|  | 398338289 | A | A | C | 1 | ANN=C |  |  |
|  | 398338296 | G | G | A | 1 | ANN=A |  |  |
|  | 398338964 | C | C | T | 1 | ANN=T |  |  |
|  | 398339033 | T | T | C | 1 | ANN=C |  |  |
|  | 398339087 | A | A | G | 1 | ANN=A | **missense_variant** | **MODERATE** |
|  | 398339088 | C | C | A | 1 | ANN=C |  |  |
|  | 398339139 | G | G | T | 1 | ANN=G |  |  |
|  | 398339439 | C | C | T | 1 | ANN=C | downstream_gene_variant | **MODIFIER** |
|  | 398340409 | T | T | C | 1 | ANN=T | 3_prime_UTR_variant |  |
|  | 398341093 | T | T | A | 1 | ANN=T |  |  |
| ***Ubiquitin carboxyl-terminal hydrolase (Lcu.2RBY.3g067540)*** | 399097610 | T | T | G | 1 | ANN=T | 5_prime_UTR_variant |  |
|  | 399098631 | G | G | A | 1 | ANN=G | **intron_variant** |  |
|  | 399099562 | A | A | G | 1 | ANN=A |  |  |
|  | 399099834 | A | A | G | 1 | ANN=A |  |  |
|  | 399100118 | G | G | T | 1 | ANN=G |  |  |
|  | 399101882 | T | T | A | 1 | ANN=T | missense_variant&splice_region_variant | **MODERATE** |
|  | 399104284 | C | C | T | 1 | ANN=C | **intron_variant** | **MODIFIER** |
|  | 399104599 | T | T | C | 1 | ANN=T |  |  |
|  | 399104609 | A | A | T | 1 | ANN=A |  |  |
|  | 399105055 | T | T | C | 1 | ANN=T |  |  |
|  | 399105138 | A | A | T | 1 | ANN=A |  |  |
|  | 399105423 | T | T | C | 1 | ANN=T | missense_variant | **MODERATE** |
|  | 399105469 | G | G | A | 1 | ANN=G | synonymous_variant | **LOW** |
|  | 399106272 | T | T | A | 1 | ANN=T | **intron_variant** | **MODIFIER** |
|  | 399106336 | T | T | G | 1 | ANN=T |  |  |
|  | 399107084 | A | A | G | 1 | ANN=A | **3_prime_UTR_variant** |  |
|  | 399107166 | T | T | A | 1 | ANN=T |  |  |
|  | 399107169 | T | T | A | 1 | ANN=T |  |  |
|  | 399107440 | C | C | T | 1 | ANN=C |  |  |
|  | 399107445 | A | A | G | 1 | ANN=A |  |  |
|  | 399107493 | A | A | T | 1 | ANN=A |  |  |
|  | 399107552 | T | A | T | 1 | ANN=A |  |  |
|  | 399107728 | G | G | C | 1 | ANN=G |  |  |
|  | 399107783 | A | A | G | 1 | ANN=A |  |  |
|  | 399107826 | A | A | T | 1 | ANN=A |  |  |
|  | 399108043 | T | T | C | 1 | ANN=T |  |  |
|  | 399108044 | G | G | A | 1 | ANN=G |  |  |
|  | 399108246 | G | G | T | 1 | ANN=G | **downstream_gene_variant** |  |
|  | 399108269 | C | C | T | 1 | ANN=C |  |  |
|  | 399108270 | T | T | C | 1 | ANN=T |  |  |
